# Label-free autofluorescence lifetime reveals the structural dynamics of ataxin-3 inside droplets formed via liquid–liquid phase separation

**DOI:** 10.1038/s41598-023-33268-y

**Published:** 2023-04-19

**Authors:** Uchu Matsuura, Shinya Tahara, Shinji Kajimoto, Takakazu Nakabayashi

**Affiliations:** 1 Graduate School of Pharmaceutical Sciences, Tohoku University, Sendai, 980-8578 Japan; 2 JST PRESTO, Kawaguchi, Saitama 332-0012 Japan

**Keywords:** Biological fluorescence, Protein aggregation, Protein aggregation, Biophysical chemistry

## Abstract

Liquid–liquid phase separation is a phenomenon that features the formation of liquid droplets containing concentrated solutes. The droplets of neurodegeneration-associated proteins are prone to generate aggregates and cause diseases. To uncover the aggregation process from the droplets, it is necessary to analyze the protein structure with keeping the droplet state in a label-free manner, but there was no suitable method. In this study, we observed the structural changes of ataxin-3, a protein associated with Machado–Joseph disease, inside the droplets, using autofluorescence lifetime microscopy. Each droplet showed autofluorescence due to tryptophan (Trp) residues, and its lifetime increased with time, reflecting structural changes toward aggregation. We used Trp mutants to reveal the structural changes around each Trp and showed that the structural change consists of several steps on different timescales. We demonstrated that the present method visualizes the protein dynamics inside a droplet in a label-free manner. Further investigations revealed that the aggregate structure formed in the droplets differs from that formed in dispersed solutions and that a polyglutamine repeat extension in ataxin-3 hardly modulates the aggregation dynamics in the droplets. These findings highlight that the droplet environment facilitates unique protein dynamics different from those in solutions.

## Introduction

Liquid–liquid phase separation (LLPS) is a phenomenon in which homogeneous polymer solutions separate into multiple liquid phases with different solute concentrations. In biology, LLPS has been adopted to describe various intracellular events^1–3^. Biomolecules such as nucleic acids and proteins undergo LLPS and form liquid droplets containing highly concentrated solutes. The formation of liquid droplets of specific biomolecules facilitates cellular functions, including stress responses^4–6^, transcription^7–10^, and metabolic reactions^11,12^. Reversibility is an important facet of LLPS that regulates intracellular reactions. For example, the droplets in a cell formed in response to hyperosmotic stress dissipate when the stress is removed^5^. The formation and dissipation of the droplets also occur in response to changes in thermodynamic parameters such as temperature and pressure.^13,14^.

While LLPS plays pivotal roles in regulating physiological functions, it is intimately related to neurodegenerative diseases^15–17^, cancers^18^, and SARS-CoV-2^19^. Various neurogenerative diseases such as amyotrophic lateral sclerosis, Alzheimer’s disease, and Parkinson’s disease have been linked with aggregation of fused in sarcoma, TDP-43, Tau, and α-Synuclein^20,21^. These proteins were recently shown to undergo LLPS^22–28^, and the generated protein droplets have been proposed to be the precursors of irreversible aggregation. Elucidating the molecular mechanism by which LLPS promotes protein aggregation is required to understand the onset mechanism of neurodegenerative diseases and develop effective treatments.

Machado–Joseph disease (MJD) is a neurodegenerative disease and common autosomal dominant spinocerebellar degeneration worldwide^29,30^. The symptoms include ataxic symptoms, peripheral nerve damage, and pyramidal tract dysfunction. The pathogenesis of MJD is believed to involve an abnormal aggregation of the protein, ataxin-3. Ataxin-3 functions as a deubiquitinating enzyme and is composed of an enzyme domain called the Josephin domain (JD), ubiquitin interaction motifs (UIM), and a polyglutamine repeat (polyQ) at the C-terminal (Fig. S1). MJD is known to be caused by an aberrant increase in the polyQ length, caused by an overextension of the number of CAG repeats in the gene encoding ataxin-3. MJD belongs to a group of diseases called “polyglutamine diseases” caused by an abnormal polyQ length^31^. Ataxin-3 is regarded as pathogenic when the polyQ exceeds ~40 residues^32^.

Ataxin-3, normally distributed in the cytoplasm, forms insoluble aggregates in the nuclei of neurons in MJD patients and is thought to cause cell dysfunction and death^29,30^. The elongation of polyQ is involved in promoting ataxin-3 aggregation and in the pathogenesis of MJD. A previous study proposed a two-stage model to explain the polyQ length dependence of the ataxin-3 aggregation^33^. In the first stage, reversible fibrillation occurs in a polyQ-independent manner. The second stage is observed only for ataxin-3 with an extended polyQ and generates stable fibrils irreversibly. In a previous study, we observed that ataxin-3 with a 28-residue polyQ (Q28) underwent LLPS, while that without a polyQ did not exhibit LLPS^34^, suggesting the mechanism of ataxin-3 aggregation via droplet formation. However, the molecular mechanism by which the polyQ elongation causes aggregation remains unknown.

Various experimental techniques have been applied to investigate the properties and structures of proteins inside droplets^35^. Fluorescence labelling has been employed to examine protein structures inside droplets. For example, the K18 fragment of Tau inside the droplets was found to exist as a more extended structure than that in an aqueous solution using the excimer fluorescence of pyrene attached to the protein^36^. However, fluorescence labelling alters weak intermolecular interactions between proteins^37–41^, resulting in changes in the properties of the droplets. NMR and EPR were used to investigate the protein structure inside the droplets^25,42,43^; however, it cannot be applied to a single liquid droplet because of its limited spatial resolution. The time evolution of droplets is difficult to be measured by NMR and EPR. Raman spectroscopy was employed to quantify the protein concentration in a single droplet in a label-free manner^34,44,45^ and was recently used to observe the secondary structural changes in the C-terminal domain of TDP-43 associated with fibril formation^46^. However, the Raman signal is not always sensitive to slight changes in the molecular structure. Accordingly, information on the structural changes in ataxin-3 with the droplet formation was not obtained in our previous Raman study^34^. The development of label-free microscopic methods is in high demand to elucidate the intrinsic properties of proteins inside droplets.

Autofluorescence is fluorescence from fluorescent molecules or moieties intrinsically present in proteins and cells. Autofluorescence has long been used to investigate structures of proteins, ligand binding to proteins, and states of cells^47–54^. In particular, protein structures can be sensitively monitored by the fluorescence of tryptophan (Trp) residues embedded in the protein, and Trp fluorescence is widely used to clarify changes in the protein structure, such as denaturation^47,49^. Protein structural changes cause environmental changes around Trp residues, such as hydrophobicity, leading to changes in their fluorescence spectra and lifetimes. The intensity of autofluorescence is weaker than that of typical fluorescent tags. Thus, lifetime measurements, which are more quantitative than intensity measurements, are used to analyze autofluorescence. Autofluorescence lifetime measurements can readily be extended to microscopy^50,55^.

In this study, we used the autofluorescence of the Trp residues of ataxin-3 to capture protein conformational changes within a single droplet. Ataxin-3 contains three Trp residues, and we observed that both the lifetimes and spectra of the autofluorescence of these Trp residues changed upon LLPS and during the subsequent aggregation process. These changes in the autofluorescence reflected the protein structural changes in the droplet, and the use of several Trp mutants enabled us to reveal the multistep changes in ataxin-3 during the phase transition from the droplets to the aggregates. We proposed that autofluorescence lifetime enables label-free observations of the dynamics of proteins inside droplets formed via LLPS. We also compared the aggregation dynamics of ataxin-3 with extended and non-extended polyQs. We found that both ataxin-3 proteins show similar aggregation time courses in the droplets, which is different from the polyQ dependence of the aggregation dynamics in dispersed solutions.

## Experimental methods

### Preparation of Q28 and Q64

The preparation of Q28 and its mutants were previously described^34^. Q28 with an N-terminal hexahistidine tag was overexpressed in an *Escherichia coli* BL21(DE3) strain (Agilent). The obtained proteins were purified by Ni–NTA chromatography (HisTrap HP, Cytiva), followed by size exclusion chromatography (HiPrep 26/60 Sephacryl S-100 HR and ӒKTAPrime Plus, Cytiva). The same protocol was used to prepare Q64 (ataxin-3 with a 64-residue polyQ).

### Purification of PEG

PEG6000 (Nacalai Tesque) was dissolved in hot ethanol, and hexane was added to induce the precipitation of PEG6000. After that, the precipitated PEG6000 was then washed with hexane and diethyl ether, dried and dissolved in distilled water. The obtained aqueous solution of PEG6000 was dialyzed against distilled water for approximately a week and lyophilized.

### LLPS and incubation of Q28 and Q64

50 mM phosphate buffer containing 150 mM NaCl at pH 7.4 (buffer A) was used as the buffer for Q28 and Q64. The concentrations of Q28 and Q64 were evaluated using the absorbance at 280 nm (ε_280_ = 28,795 mol^−1^ cm^−1^, U-3300, Hitachi) and adjusted to 200 μM using centrifugal filters (Amicon Ultra-15, Merck Millipore). The same buffer was used to prepare purified 40 w/w% PEG solutions. An equal volume of the 200-μM Q28 or Q64 solution was mixed with 40 w/w% PEG by pipetting. The same protocol was used for LLPS of the mutants. The formation of the droplets was confirmed by an inverted microscope (Eclipse Ti-U, Nikon). The droplets were incubated at 296 K under humidity of 100% to alleviate sample evaporation.

### Fluorescence lifetime imaging

We constructed a fluorescence lifetime imaging system equipped with a light-emitting diode (Fig. S2), which provided UV-pulsed light at 299 nm with a 500-ps duration at a 10-MHz repetition rate (PLS-290–10, PicoQuant GmBH). The polarization of the excitation light was set to horizontal using a wire-grid polarizer (WP25-UB, Thorlabs). The excitation light was subsequently introduced into an inverted microscope (Axiovert 135, Zeiss) equipped with a dichroic mirror (Di01-R325-25 × 36, Semrock) and a UV-enhanced reflective objective with a magnification of 40 (LMM40X-UVV, Thorlabs). A plano-convex lens (f = 200 mm) was introduced just before the microscope to achieve a wide-field illumination at the focal plane. The obtained fluorescence signal was introduced into a band-pass filter (12–096, Edmund Optics) and a wire-grid polarizer (WP25-UB, Thorlabs) set to the magic angle with respect to the polarization of the excitation light. The fluorescence was focused onto a two-dimensional sensor of a disk anode microchannel plate photomultiplier tube (PSC-01, Biomedical Instruments) to obtain fluorescence decay curves at each spatial point based on the time-correlated single-photon counting (TCSPC) method. The fluorescence intensity image was constructed by integrating the fluorescence decay curve at each spatial point. Fluorescence decay curves of the mutants were also measured using a photomultiplier tube for TCSPC (PMC-150–210, Becker & Hickl GmbH). Bright-field images were obtained using a CCD camera (DP70, Olympus).

### Measurements of fluorescence spectra

Fluorescence spectra were measured using a fluorophotometer (FP-6500, Jasco) and a quartz cuvette with a 3-mm path length (3–3.45Q/3, Starna). The excitation wavelength was 299 nm.

### Measurements of circular dichroism spectra

Circular dichroism spectra were measured using a spectropolarimeter (J-820, Jasco) and a quartz cuvette with a 0.5-mm path length (S10-UV-0.5, GL Sciences).

### Thioflavin T (ThT) fluorescence measurements

100-μM solutions of Q28 and its droplets were incubated at 310 K for 10 days and at 296 K for 2 days, respectively. The 20-μL sample solutions were mixed with 20-μL, 300-μM ThT dissolved in buffer A and 160-μL buffer A. Then, fluorescence spectra were measured using a fluorophotometer (FP-6500, Jasco). The excitation wavelength was 430 nm.

## Results and discussion

**Figure 1:**
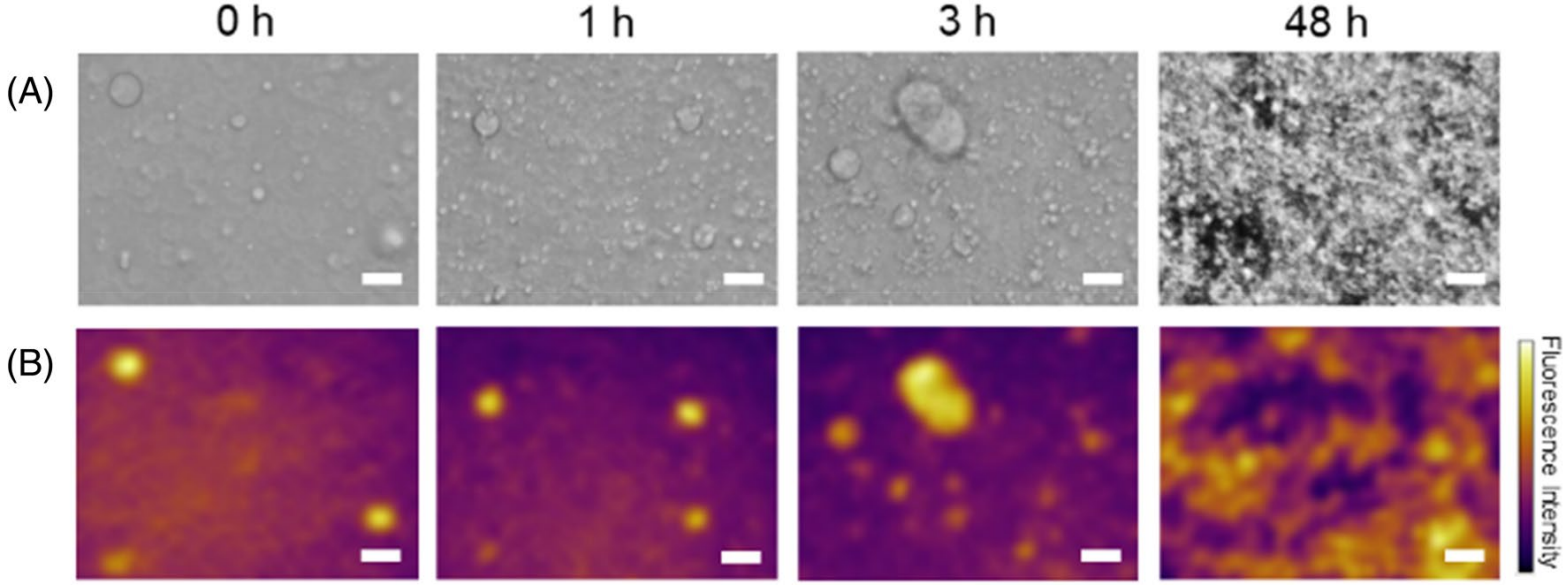
(**A**) Bright-field and (**B**) autofluorescence images of Q28 droplets at incubation times of 0, 1, 3, and 48 h. Scale bar = 20 μm.

The formation of the Q28 droplets with a diameter in the range of 10–20 μm was confirmed in bright-field images after adding the PEG solution (which mimicked the intracellular molecular crowding environment) to the Q28 solution (Fig. 1A). The droplets formed via LLPS were incubated at 296 K. The shape of the droplets was spherical at 1 h after LLPS and changed after further incubation. The droplet after the 3-h incubation showed spherical and distorted structures, implying a liquid-to-solid phase transition of the Q28 droplets. After 48 h, the protein aggregates were dominantly observed.

We acquired autofluorescence images of the droplets and aggregates of Q28 (Fig. 1B) using the constructed UV-excited microscope system (Fig. S2). The droplets exhibited strong autofluorescence with the 299-nm excitation, indicating that Q28 was highly condensed inside the droplets. This conclusion is supported by our previous study, which showed that the concentration of Q28 within the droplets was 13.6 mM, which was over a thousand times larger than that outside the droplets (~ 6 μM).^34^ The present study demonstrates that droplets formed via LLPS can be visualized in a label-free manner using autofluorescence.

The observed autofluorescence is primarily attributable to the Trp residues. Ataxin-3 possesses three Trp residues, eight tyrosine residues, and ten phenylalanine residues. The molar coefficient of Trp at the excitation wavelength was overwhelmingly higher than those of the tyrosine and phenylalanine residues (Fig. S3); thus, the Trp residues were selectively excited and dominantly contributed to the autofluorescence signals. Impurities in crude PEG6000 exhibited background fluorescence, and in the present study, the fluorescent impurities were removed by the purification mentioned in the Experimental methods section. The fluorescence of the solution containing only PEG6000 was negligibly weak after the purification compared with that of the Trp residues of Q28 (Fig. S4).

We then constructed an autofluorescence lifetime image of the Q28 droplets immediately after LLPS (Fig. S5). Although the precision of the calculated lifetimes is limited due to the low S/N ratios of the fluorescence decay curves, we observed no specific spatial distribution of the lifetime inside the droplets. We then compared autofluorescence decay curves of individual droplets immediately after LLPS (Fig. S6). To improve the S/N ratio, we summed up the fluorescence decay curves at multiple spatial points inside the droplets. Each droplet showed no significant difference in the decay profile, suggesting no marked variation in the protein structure among the droplets.

The areas outside the droplets also showed fluorescence signals much weaker than those inside the droplets. To clarify the origin of the signals outside the droplets, we compared the fluorescence decay curves inside and outside the droplets. The shapes of these decay profiles were essentially identical (Fig. S7), indicating that the fluorescence observed outside the droplets is originated from the droplets that were out of the focal plane of the objective. This result is consistent with the very high concentration of Q28 inside the droplets mentioned above. Hereafter, the autofluorescence decay curves were obtained by summing up the decay curves at all the pixels in the image, and they were used for the following analyses.

**Figure 2:**
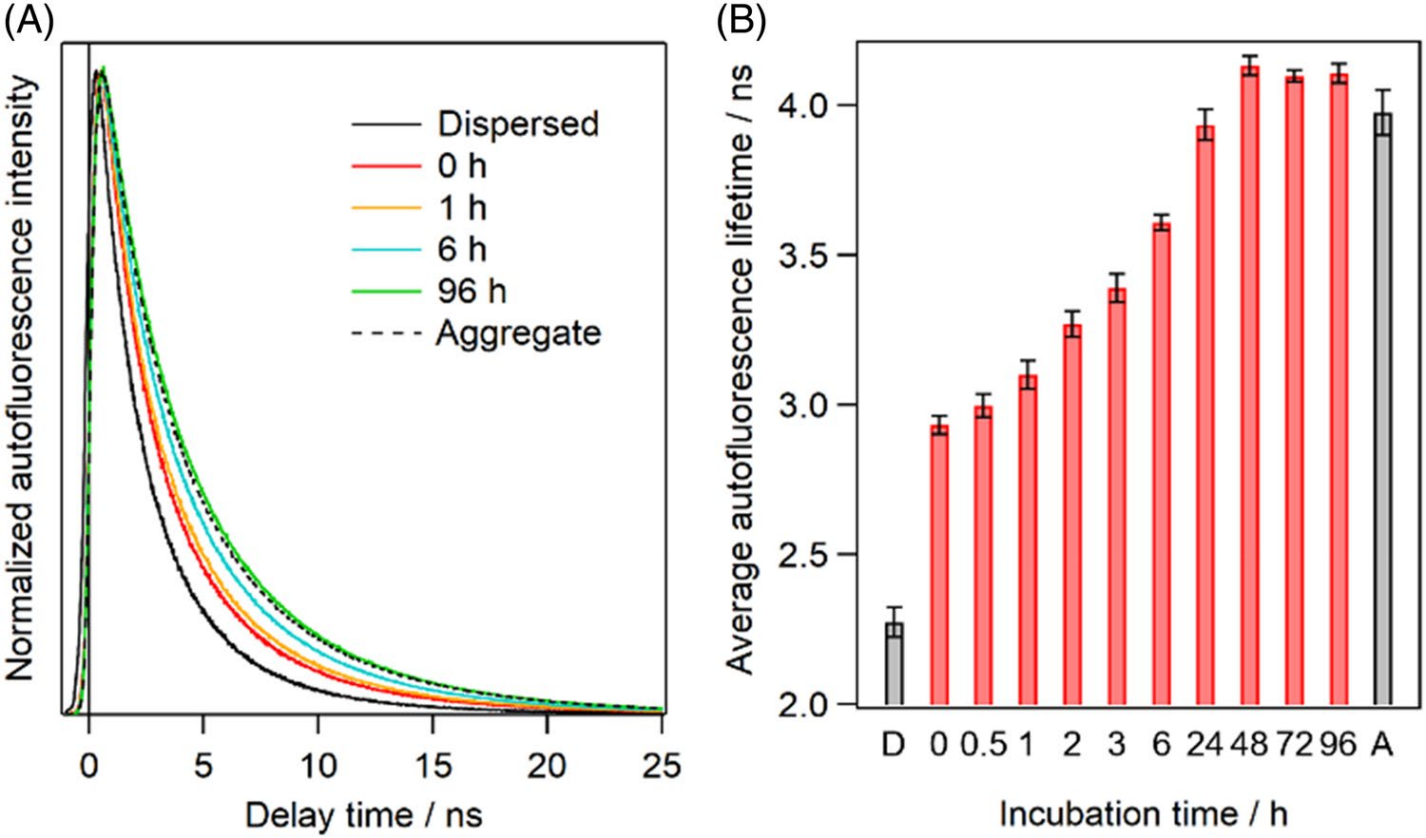
(**A**) Normalized autofluorescence decay curves and (**B**) average autofluorescence lifetimes of Q28 in the dispersed solution, the droplets, and the aggregates. In (**A**), coloured solid curves are the autofluorescence decay curves of the droplets at various incubation times. Black solid and broken curves are the autofluorescence decay curves of the dispersed solution and the aggregates, respectively. In (**B**), red bars are the average lifetimes of the droplets at various incubation times. Values below the bars are the incubation times in hours. “D” and “A” below the bars represent the dispersed solution and the aggregates, respectively. Error bars are SD (The numbers of replicates are summarized in Table S1).

We measured autofluorescence decay curves of the Trp residues of Q28 in the droplets at various incubation times. We also measured the decay curves of Q28 dispersed in the buffer solution (dispersed solution) and the solid aggregates directly formed from the dispersed solution as the controls to detect unique features of Q28 inside the droplets (Fig. 2A). The formation of the aggregates directly from the dispersed solution required an incubation condition harsher (at 310 K for 48 h) than that via LLPS, suggesting that the droplet environment promotes the aggregation. The autofluorescence of the droplets after 0 h (measured immediately after preparation of the droplets) decayed more slowly than that of the dispersed solution. The observed slower decay of the droplets reflects the unique solvent environment and protein structure inside the droplets. Effects of adding PEG on the Trp autofluorescence inside the droplets can be neglected because the PEG concentration inside the droplets is very limited (< 1 wt%)^34^. We also found that adding 5% PEG to the Q28 dispersed solution rather slightly accelerated the autofluorescence decay (Fig. S8), opposite to the observation of the slower decay in the droplets. The autofluorescence decay became slower with an increase in the incubation time. After the 96-h incubation, the autofluorescence decay curve of the droplets almost coincided with that of the aggregates prepared from the dispersed solution.

We performed fitting analyses of the autofluorescence decays to examine the time evolution of the proteins inside the droplets. The data points before 500 ps showed the distortion of the curves due to the finite time resolution, and thus were not used for the analyses. The curves could not be fitted with a single exponential function but well fitted with the sum of three exponential decay functions convoluted with a Gaussian function whose width was determined by fitting the instrumental response function (Fig. S9). The largest time constant was fixed at 10 ns to reproduce the weak but prolonged fluorescence decay observed for all the samples. It has been reported that Trp fluorescence shows a nonexponential decay, and the consensus on the origin of this unusual decay has yet to be reached. We calculated the average autofluorescence lifetime (τ_ave_) to quantitatively analyze the changes in the autofluorescence decay curve (Fig. 2B). The sum of the lifetimes (τ_i_) of the three components weighted by their pre-exponential factors (*C*_i_) was used as the average autofluorescence lifetime as follows.

1 $${\tau }_{ave}= \begin{array}{c}\frac{{\displaystyle\sum_{\ i=1}^{3}{C}_{i}{\tau }_{i}}} {\displaystyle\sum_{\ i=1}^{3}{C}_{i}}\end{array}$$

The average autofluorescence lifetime of the droplets with 0 h incubation was markedly more prolonged than that of the dispersed solution. This result indicates that the environment around the Trp residues of Q28 in the droplets differs from that in the dispersed solution. The average lifetime of the droplets increased with incubation time and approached that of the aggregates after the incubation for 48 h. These observations reflect changes in the environment surrounding the Trp residues of Q28, originating from the structural changes in Q28 inside the droplets associated with the aggregation.

**Figure 3:**
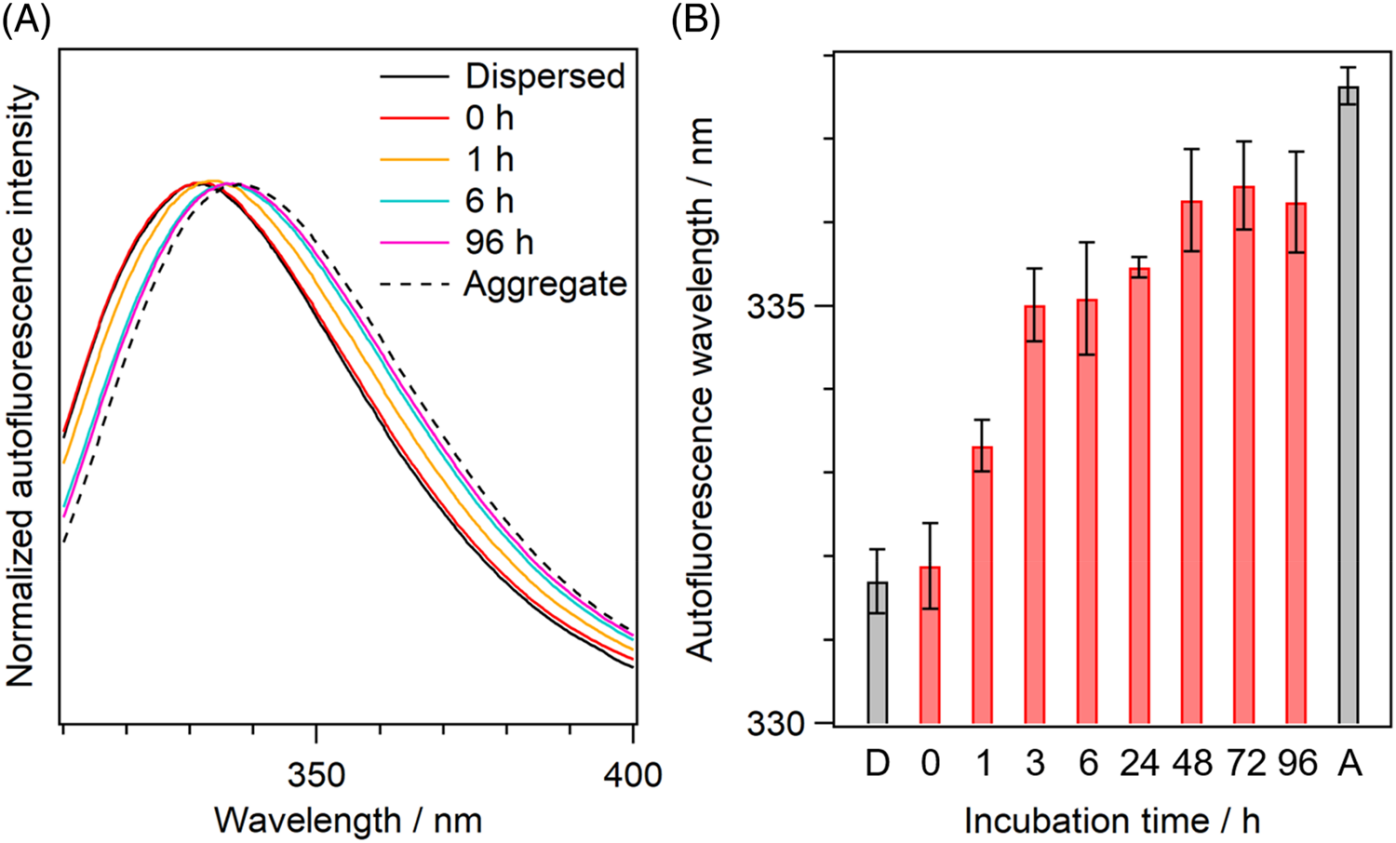
(**A**) Normalized autofluorescence spectra and (**B**) their peak wavelengths of Q28 in the dispersed solution, the droplets, and the aggregates. In (**A**), coloured solid curves are the autofluorescence spectra of the droplets at various incubation times. Black solid and broken curves are the autofluorescence spectra of the dispersed solution and the aggregates, respectively. In (**B**), red bars are the peak wavelengths of the droplets at various incubation times. Values below the bars are the incubation times in hours. “D” and “A” below the bars represent the dispersed solution and the aggregates, respectively. Error bars are SD (The numbers of replicates are summarized in Table S1).

To clarify the specific environmental changes that occurred around the Trp residues, we measured the autofluorescence spectra of the Trp residues of Q28 in the droplets at different incubation times, along with the dispersed solution and solid aggregate (Fig. 3A). The autofluorescence spectrum of the droplets at 0 h incubation time coincided with that of the dispersed solution. The spectra of the droplets showed a gradual red shift with increasing incubation time, and the aggregates exhibited the longest autofluorescence wavelength (Fig. 3B). It is known that the peak wavelength of Trp fluorescence positively correlates with the hydrophilicity of the surrounding environment, which comes from the significant increase in the dipole moment of Trp in the fluorescence state. Thus, the observed spectral changes indicate that the environments of the Trp residues of Q28 in the droplets become hydrophilic with incubation. The aggregates show the longest peak wavelength, indicating that the hydrophilicity around the Trp residues is the highest among the measured conditions.

**Figure 4:**
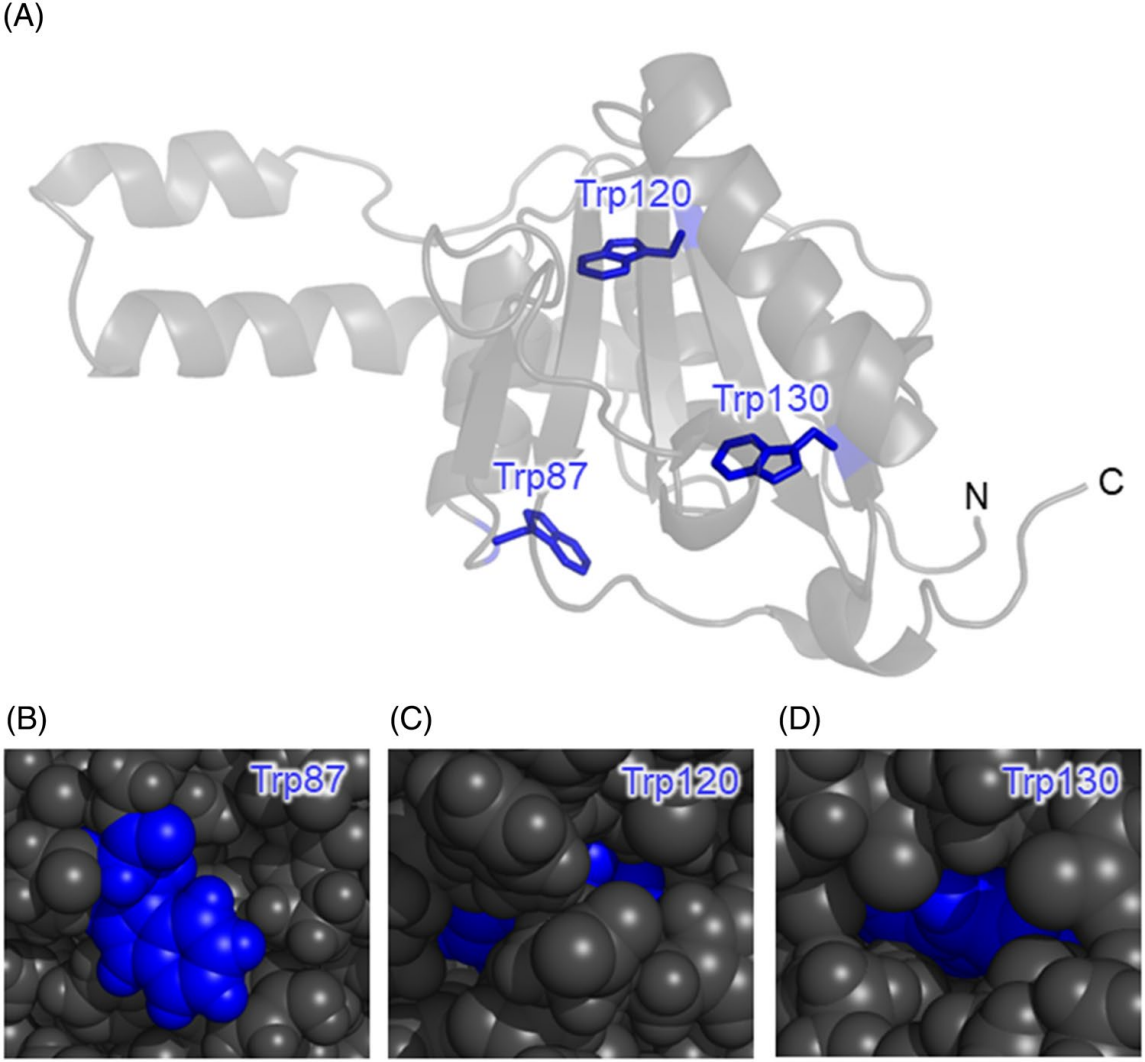
Solution NMR structure of JD of ataxin-3 (PDB code: 1YZB). (**A**) Three Trp residues, Trp87, Trp120, and Trp130 with blue sticks. (**B**–**D**) Magnified views of the protein structures around these Trp residues with van der Waals surface representation.

We showed using the autofluorescence that the environment around the Trp residues changes to hydrophilic during the transition from the droplets to the aggregates. Next, we examined how the three Trp residues in Q28 become hydrophilic using Trp mutants. Q28 possesses three Trp residues, Trp87, Trp120, and Trp130, all of which exist in JD^56^ (Fig. 4A). Trp87 is located on the protein exterior and exposed to the bulk solvent (Fig. 4B), while Trp120 and Trp130 are buried in the protein (Fig. 4C,D). We substituted two of the three Trp residues with phenylalanine to prepare Q28 mutants with only a single Trp residue, W120F/W130F, W87F/W130F, and W87F/W120F.

**Figure 5:**
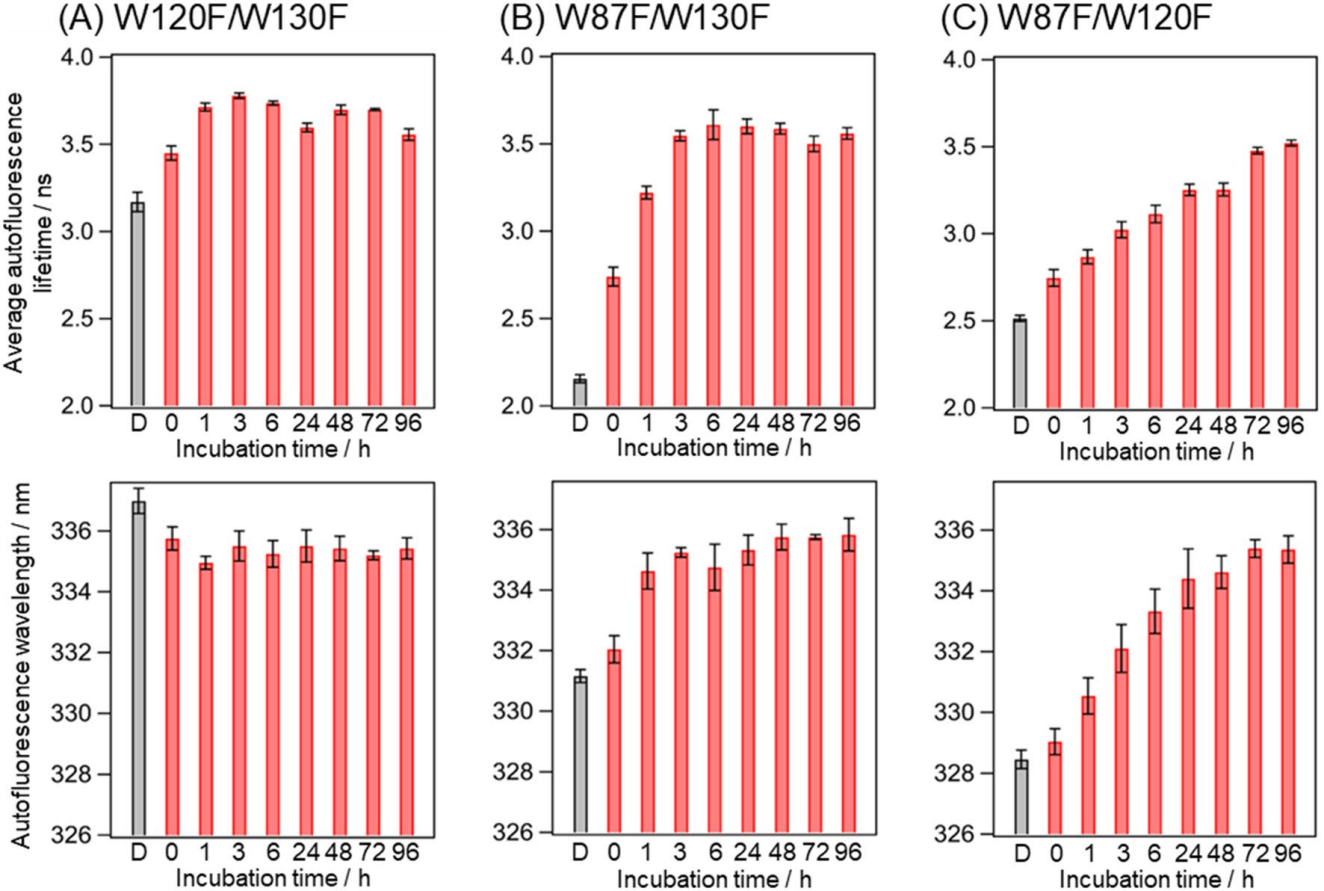
Average autofluorescence lifetimes and peak wavelengths of the autofluorescence spectra of (**A**) W120F/W130F, (**B**) W87F/W130F, and (**C**) W87F/W120F mutants of Q28 in the dispersed solution and droplets at various incubation times. Values below the bars are incubation times in hours. “D” below the bar indicates the dispersed solution. Error bars are SD (The numbers of replicates are summarized in Table S1).

The autofluorescence decay curves and spectra of the three mutants in the dispersed solution and droplets were measured (Fig. S10), and the average autofluorescence lifetime and the peak wavelength were plotted against the incubation time (Fig. 5). The autofluorescence wavelengths of Trp87, Trp120, and Trp 130 of Q28 in the dispersed solution were 336, 332, and 329 nm, respectively, indicating that the surrounding environment around Trp87 is more hydrophilic than those around Trp120 and Trp130. This tendency of the hydrophilicity is consistent with the positions of the Trp residues in the native state (Fig. 4A), confirming that the mutants retain the native protein structure. All the mutants showed a difference in the average autofluorescence lifetime between the dispersed solution and the droplets at 0 h incubation time, indicating that LLPS altered the surrounding environment of all the Trp residues. Furthermore, each mutant exhibited a distinct temporal change in autofluorescence.

Environmental changes around Trp87 can be monitored from the change in the autofluorescence of W120F/W130F. The peak wavelength of Trp87 was the longest among the three mutants in the dispersed solution (Fig. 5A). This result arises from the fact that the environment around Trp87 is hydrophilic in the dispersed state due to its water-exposed position (Fig. 4B). The autofluorescence spectrum of Trp87 exhibited a blue shift by LLPS and then remained constant with incubation time. This result indicates that LLPS causes a decrease in hydrophilicity around Trp87, which then does not change over time; the decrease in hydrophilicity caused by LLPS indicates that the environment inside the droplets is more hydrophobic than water. The inside of the droplet is considered more like an organic solvent than water because of the high protein concentration.

Environments around buried Trp120 and Trp130 were examined using the autofluorescence of W87F/W130F and W87F/W120F, respectively. A red shift was observed for Trp120 and Trp130 upon LLPS (Fig. 5B,C), reflecting the increase in the hydrophilicity around Trp120 and Trp130. These mutants exhibited a further red shift with incubation. The rate of the wavelength change differed between these two mutants, and the red shift of Trp120 was completed within 3 h, while that of Trp130 took approximately 24 h. These observations indicate that the hydrophilicity around Trp120 and Trp130 increases with the incubation on different timescales. After the aggregation (> 48 h), the peak wavelengths of Trp120 and Trp130 were close to that of Trp87 (~ 335 nm). This means that the degree of the hydrophilicity around all the three Trp residues was shown to be comparable in the aggregate. This wavelength is considerably shorter than that of Q28 after denaturation with guanidinium chloride (~ 352 nm, Fig. S11) whose Trp residues are entirely exposed to water. Thus, the Trp residues are only partially exposed to water in the aggregates.

The autofluorescence peak wavelength of Trp120 and Trp130 increased with the incubation (Fig. 5B,C). This result reflects the increase in the hydrophilicity around these Trp residues, which means that the proteins inside the droplets undergo structural changes. Concomitant with the increase in the hydrophilicity, the autofluorescence lifetime increased. The –COOH, –COO^−^, –NH_3_^+^, –NH_2_, –SH, imidazole groups of the amino acids are shown to function as intrinsic quenchers of the Trp fluorescence^57^. As shown in Fig. S12, Trp120 and Trp130 are surrounded by the intrinsic quencher groups in the native state. One possible reason for the increase in the fluorescence lifetime is that the distance between these quenchers and the Trp residue increases due to the structural changes in the protein. On the other hand, the average lifetime and peak wavelength behaved differently for Trp87 (Fig. 5A); the average autofluorescence lifetime increased despite the blue shift due to the increased hydrophobicity after LLPS. The average lifetime also increased after 1 h incubation, although the peak wavelength remained constant. We thus conclude that the average autofluorescence lifetime of Trp87 reflects changes in other environmental parameters as well as hydrophilicity.

**Figure 6:**
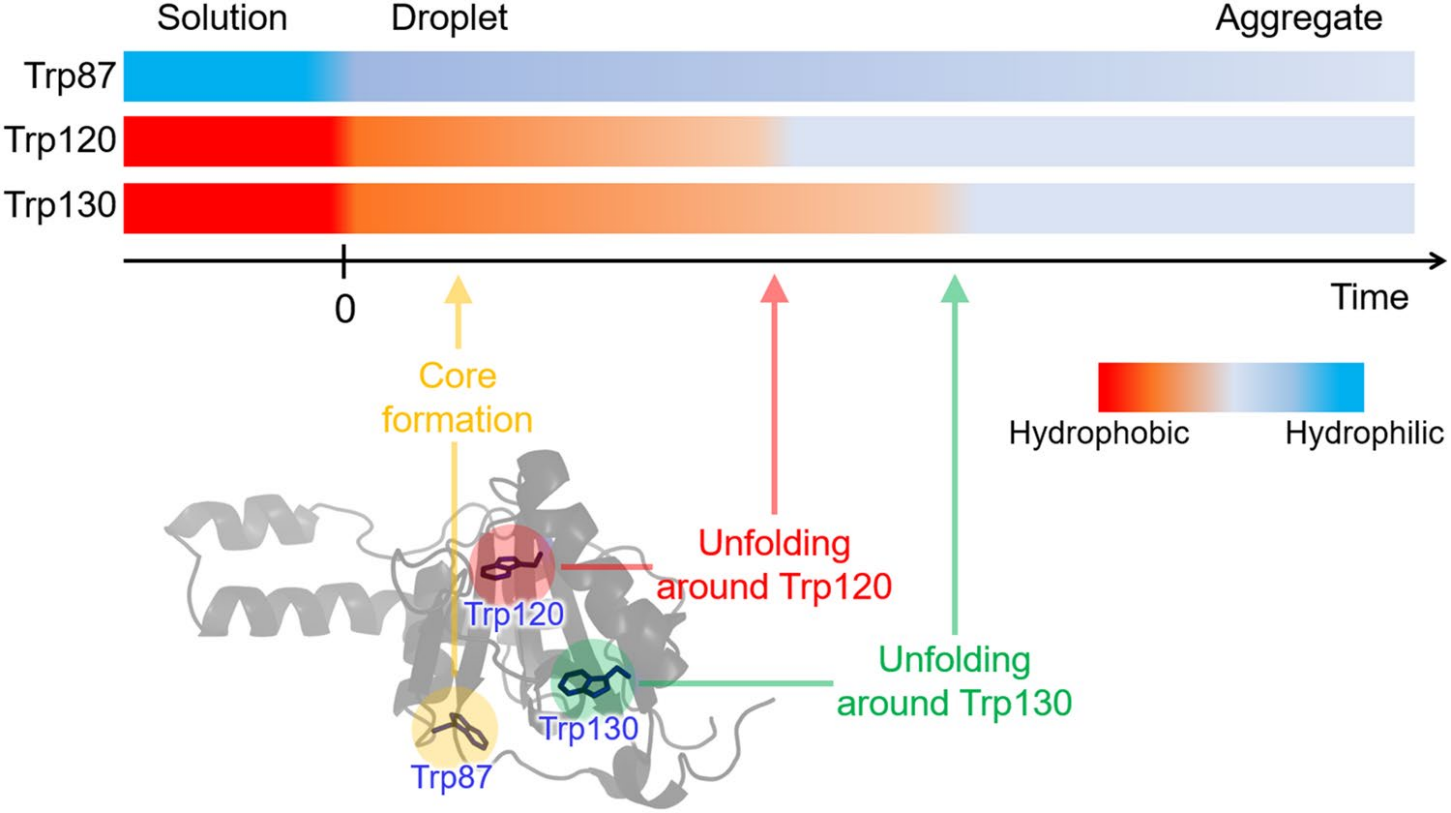
Structural changes in Q28 inside droplets and concomitant changes in the environment around the Trp residues. Horizontal bars indicated by Trp87, Trp120, and Trp130 show temporal changes in hydrophilicity around these Trp residues. LLPS occurs at time 0. The structure of JD and the arrangement of Trp residues are shown at the bottom.

We summarize the environmental changes around each Trp residue and the protein dynamics inside the droplets (Fig. 6). We observed the environment change around Trp87 within 3 h as the increase in the fluorescence lifetime, which was faster than the aggregation. This change may reflect the formation of the aggregation core. In the previous studies, the protein moiety around helix-α4 (residues 75–91) was identified as an aggregation-prone region that forms the core of the fibril in the dispersed solution^58,59^. The formation of the aggregate core may occur in the droplets and cause the changes in the surrounding environment around Trp87. The surrounding environments around buried Trp120 (Fig. 5B) and Trp130 (Fig. 5C) become hydrophilic upon LLPS. This result shows that the droplet formation induces structural changes in JD involving the opening of the cavity encapsulating both the Trp residues. The incubation of the droplets leads to the formation of the solid aggregates and further increases in the hydrophilicity around these Trp residues (Fig. 6). The structure in the resultant aggregates was investigated using circular dichroism (CD). The obtained CD spectrum exhibited a marked increase in the amplitude around 200 nm, indicating that the aggregates formed via LLPS exhibit the almost unfolded structures dominated by random coil structures (Fig. S13). The timescale of the increase in the hydrophilicity is different for the two Trp residues; Trp120 shows a faster increase than Trp130. In the case of Trp87 (Fig. 5A), environmental changes occur within 1 h. It is thus concluded that each Trp residue exhibits environmental changes on distinct timescales, indicating that the structural changes consist of several local steps. After the aggregate formation (> 48 h), the average lifetime and peak wavelength of all the mutants were nearly identical, showing that the environments around the three Trp residues were very similar in the aggregates. The Trp residues are only partially exposed to water in the aggregates, probably due to intermolecular interactions between Q28 proteins. In summary, upon LLPS, Q28 is highly concentrated in the droplets and the multistep structural changes occur, resulting in the aggregate formation.

Q28 in the aggregates via LLPS largely lost the secondary structure. On the other hand, it has been reported that ataxin-3 in buffer solutions forms amyloid-like fibrils that are β-sheet-rich aggregates^33^. This result implies that the aggregate structure formed via LLPS is different from that directly formed from the dispersed solution. To compare the aggregate structure directly formed from the dispersed solution with that formed via LLPS, we incubated Q28 at a slightly higher temperature (310 K) without inducing LLPS. The formation of the aggregate directly from the dispersed solution was confirmed after incubating at 310 K for 10 days by monitoring the autofluorescence wavelengths of each Trp residue (Fig. S14). The CD spectra indicated that a random coil structure was dominant in the aggregates formed via LLPS, whereas the aggregates directly formed from the dispersed solution had the secondary structure (Fig. S13). The fluorescence intensity of thioflavin T (ThT) was stronger for the aggregates directly formed from the dispersed solution than those formed via LLPS, suggesting that the structure of the aggregates formed via LLPS was different from the fibril aggregates formed from the dispersed solution (Fig. S15). These observations suggest that LLPS modulates the aggregation pathway of Q28. We note that the observed difference in the aggregation dynamics between the droplets and the dispersed solution was not caused by the presence of PEG. As mentioned above, our previous study showed that PEG was excluded from the droplet and the PEG concentration inside the droplets was negligible (< 1 wt%) and was not detectable by Raman microscopy^34^. We also found that the presence of 5 wt% PEG in the dispersed solution did not cause the aggregation dynamics similar to that occurred in the droplets. We incubated the dispersed solution containing 5 wt% PEG6000 at 296 K for 48 h and showed that its CD spectrum was largely different from that of the aggregates formed via LLPS (Fig. S13).

**Figure 7:**
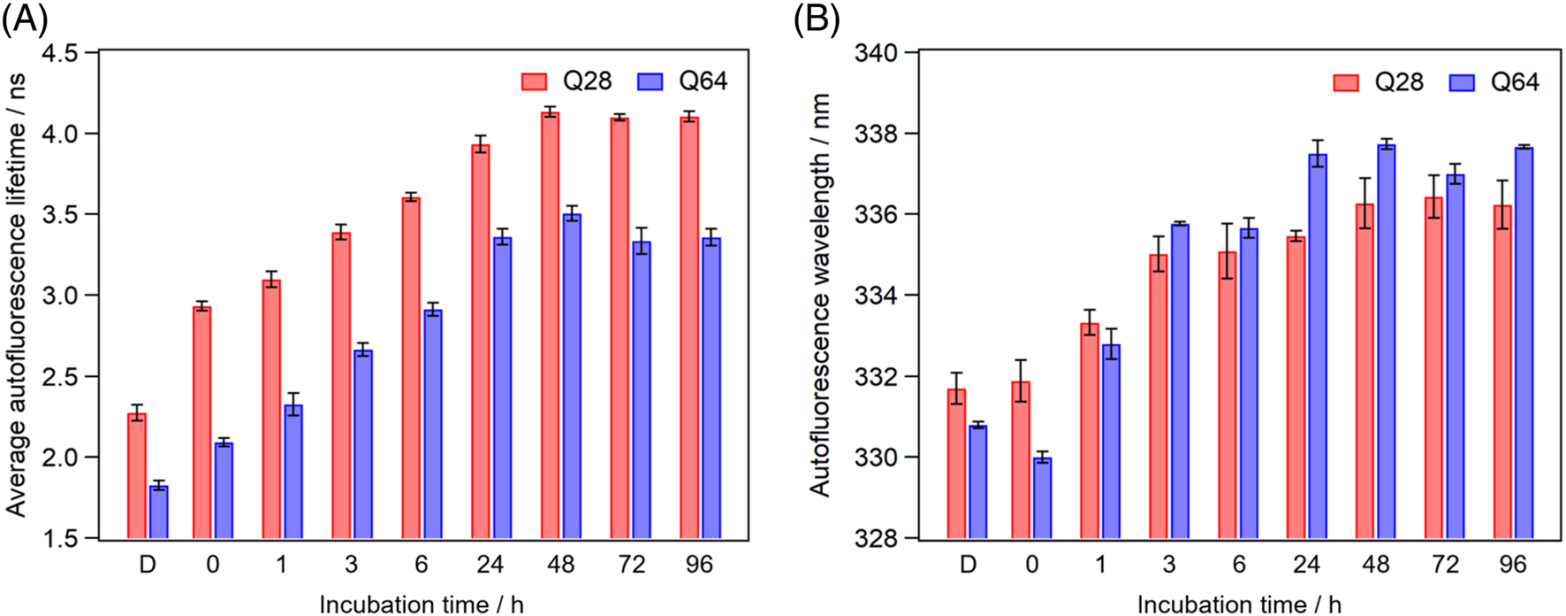
(**A**) Average autofluorescence lifetimes and (**B**) peak wavelengths of the autofluorescence spectra of Q28 and Q64 in the dispersed solution and droplets at various incubation times. Values below the bars are incubation times in hours. “D” below the bar indicates the dispersed solution. The results of Q28 were the same as those in Figs. 2B and 3B. Error bars are SD (The numbers of replicates are summarized in Table S1).

An extension of the polyQ promotes the fibrillation process in the dispersed solution^32^. We thus next compared the aggregation dynamics of Q28 and Q64 following LLPS (Fig. S16A). We succeeded in inducing LLPS of Q64 using the same method for the preparation of Q28 droplets (Fig. S16B), and compared the fluorescence lifetimes (Fig. 7A) and peak wavelengths (Fig. 7B) of Q28 and Q64 in the droplets after the incubation. The autofluorescence wavelength of Q64 decreased immediately after LLPS, indicating the hydrophobic environment in the droplets. The autofluorescence lifetime and wavelength of Q64 gradually increased with the incubation and almost reached a plateau within 48 h, which was a comparable timescale of the aggregation of Q28. There was no marked difference in the autofluorescence wavelengths of Q64 and Q28 after the 48-h incubation. These results suggest that the polyQ extension hardly changes the dynamics of ataxin-3 associated with the aggregation via the droplets. The autofluorescence lifetime of Q64 was shorter than that of Q28 in the dispersed solution and the droplets. The extended polyQ may lead to the increase in the quenching effect due to the side chain of the glutamine residues and shorten the autofluorescence lifetime of the Trp residues.

**Figure 8:**
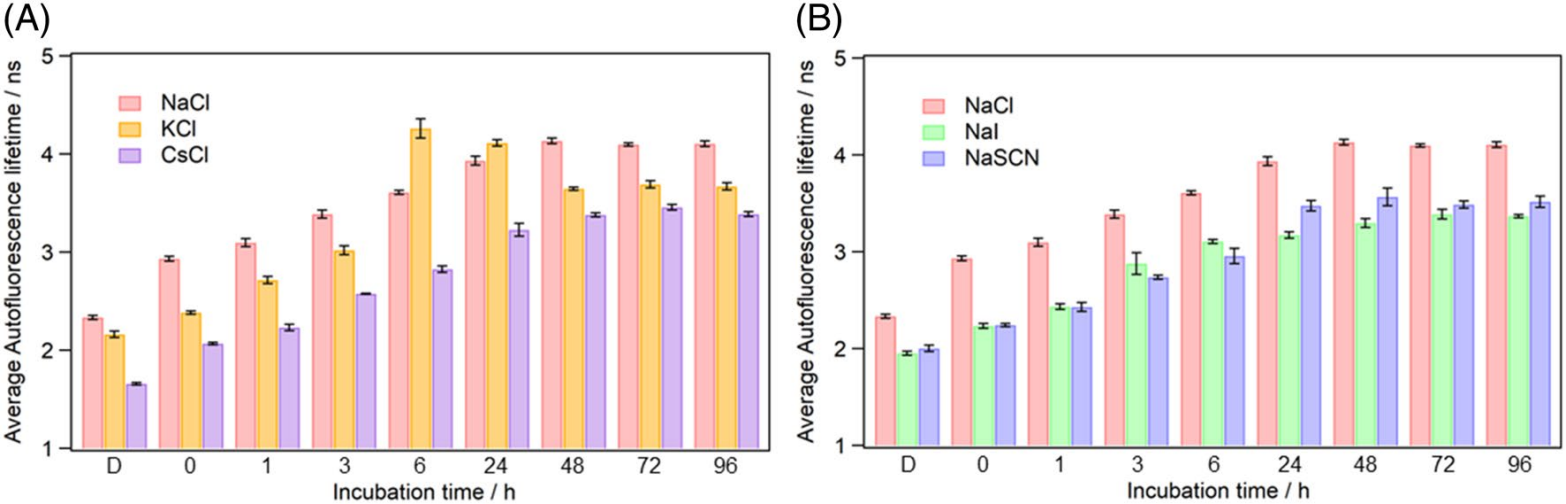
(**A**) Cation and (**B**) anion dependence of average autofluorescence lifetimes of Q28 in the dispersed solution and the droplets at various incubation time. Values below the bars are the incubation times in hours. “D” below the bar indicates the dispersed solution. Error bars are SD (The numbers of replicates are summarized in Table S1).

The present study succeeded in visualizing the structural changes in Q28 after the droplet formation. To obtain further insight into the stability of the protein inside the droplets, we investigated the salt effect on the protein structure inside the droplets. We measured the autofluorescence decay curves of the Q28 droplets in the presence of various salts and evaluated the average autofluorescence lifetimes. We compared the average lifetimes in the presence of NaCl, KCl, and CsCl to examine the effect of the cations, Na^+^, K^+^, and Cs^+^ (Fig. 8A). The increase in the average autofluorescence lifetime reflects the increase in the magnitude of the unfolding (Fig. 5). The result showed that the propensity to stabilize the protein structure decreased in the order of Cs^+^  > K^+^  > Na^+^, which is in accord with the Hofmeister series^60^. Thus, the Hofmeister effect of cations can be applied to Q28 in the droplets and aggregates formed via LLPS.

We also examined the anion dependence of the autofluorescence lifetime of Q28 inside the droplets (Fig. 8B). According to the Hofmeister series, the propensity to destabilize the protein structure decreases in the order of SCN^–^ > I^–^ > Cl^–^. The average autofluorescence lifetimes of the dispersed solution varied in the order of Cl^–^ > I^–^ ≈ SCN^–^, showing no positive correlation between the Hofmeister series of anions and the average lifetime. The observed anion dependence of the average lifetime is rationally explained by the quenching effect since I^–^ and SCN^–^ strongly quench the fluorescence of aromatic molecules, compared with Cl^−^^61^. The average lifetimes in the presence of I^−^ and SCN^−^ were shorter than that in the presence of Cl^−^ irrespective of incubation time, meaning that the anions can access the Trp residues of Q28 in the droplets and the aggregates. The quenching effect has been widely used to examine the solvent accessibility inside proteins^36^. Solvent accessibility to a protein may be altered not only by structural changes in the protein but also by changes in the hydrodynamic properties inside the droplet. Thus, it is conceivable that the quenching experiments of protein autofluorescence have the potential to provide information on phase transitions of droplets such as gelation.

The present study shows that LLPS causes Q28 to form the aggregates with almost unfolded structures. In the previous study, ataxin-3 without a polyQ region did not show LLPS under the conditions where LLPS of Q28 was observed^34^, indicating the importance of the polyQ region in LLPS. As mentioned in Introduction, aggregates of ataxin-3 with an extended polyQ were observed in patients with MJD^62^. Thus, investigating the effect of the polyQ length on the dynamics of LLPS, unfolding, and aggregation in vivo is the next step toward understanding the molecular mechanism of the onset of MJD. The present study demonstrates that autofluorescence lifetime enables the extraction of the structural information of proteins inside droplets. This method is highly promising in providing implications for the pathogenic mechanism of MJD and other neurodegenerative diseases.

## Conclusion

We demonstrated that autofluorescence lifetime and spectral measurements enable the observations of the localization and dynamics of proteins inside droplets formed via LLPS in a label-free manner. Here, we monitored the autofluorescence intensity and lifetime of the Trp residues of Q28 upon LLPS. It was confirmed that Q28 was localized inside the droplets after LLPS and that the interior of the droplets was more hydrophobic than the dispersed solution. Q28 inside the droplet was partially unfolded and finally formed the aggregates. The structural changes inside the droplets comprised several steps on different timescales. The structure of the aggregates formed via LLPS differed from that of the fibrillar aggregate directly formed from the dispersed solution, suggesting that LLPS modulates the aggregation dynamics. We also found that the aggregation dynamics of Q64 is similar to that of the Q28 aggregation inside the droplets, as contrasted with the understanding of the polyQ effect in dispersed solutions. This result highlights that the droplet environment facilitates unique protein dynamics distinct from that in dispersed solutions. Finally, autofluorescence lifetime offers a variety of information on both the microscopic and macroscopic properties of LLPS.

## Supplementary Information

Supplementary Information.

## Data Availability

All data acquired and analyzed in this study are included in this paper and the Supplementary Information file.
