# Supplementary material for: Label-free autofluorescence lifetime reveals the structural dynamics of ataxin-3 inside droplets formed via liquid–liquid phase separation
Source: Sci Rep. 2023 Apr 19;13:6389. doi: 10.1038/s41598-023-33268-y (PMC10113985; doi:10.1038/s41598-023-33268-y)
Supplement: Supplementary file 1 — Supplementary Information. [file 41598_2023_33268_MOESM1_ESM.docx]

Supplementary Information for:

Label-Free Autofluorescence Lifetime Reveals the Structural Dynamics of Ataxin-3 Inside Droplets Formed via Liquid–Liquid Phase Separation

Uchu Matsuura,^1^ Shinya Tahara,^*1^ Shinji Kajimoto,^1,2^ and Takakazu Nakabayashi^*1^

^1^Graduate School of Pharmaceutical Sciences, Tohoku University, Aoba-ku, Sendai 980-8578, Japan
E-mail: shinya.tahara.c6@tohoku.ac.jp, takakazu.nakabayashi.e7@tohoku.ac.jp

^2^JST PRESTO, Kawaguchi, Saitama 332-0012, Japan

**This PDF file includes:**

Fig. S1 to S16 and Table S1.

**
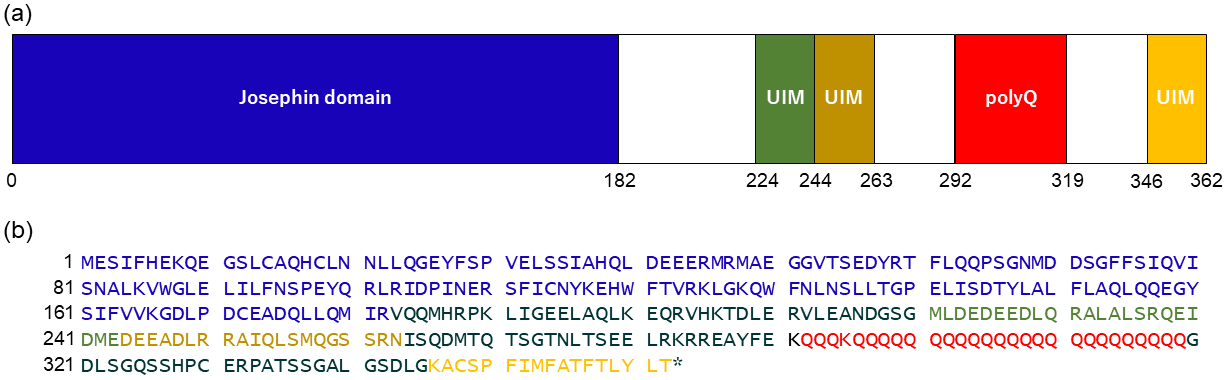
**

**Figure S1.** (a) Overview of the amino acid sequence of Q28. UIM: Ubiquitin interaction motif, polyQ: polyglutamine repeat. (a) The amino acid sequence of Q28. Josephin domain and polyQ regions are shown in blue and red, respectively. UIM regions are also shown in green, brown, and orange.

**
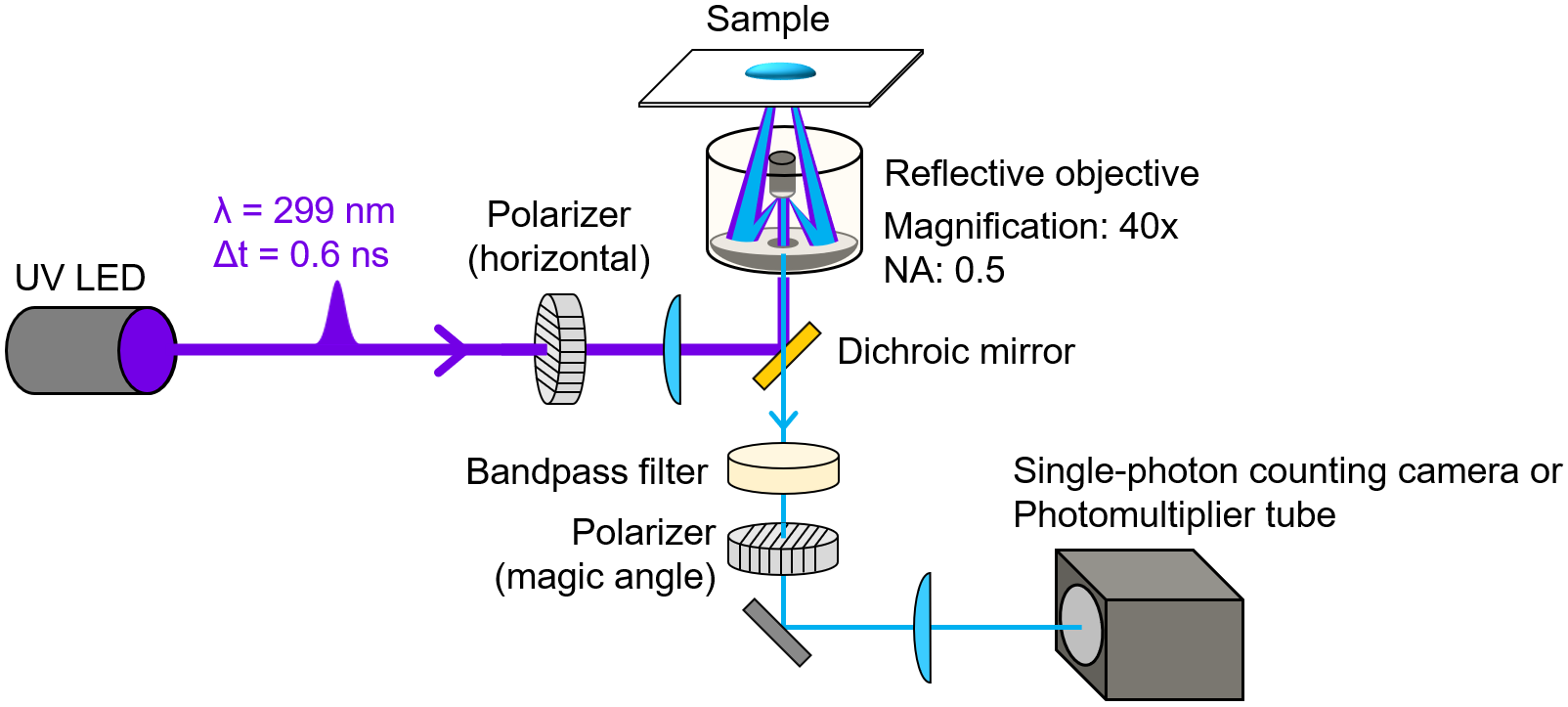
**

**Figure S2.** Schematic of UV-excited fluorescence lifetime imaging system.


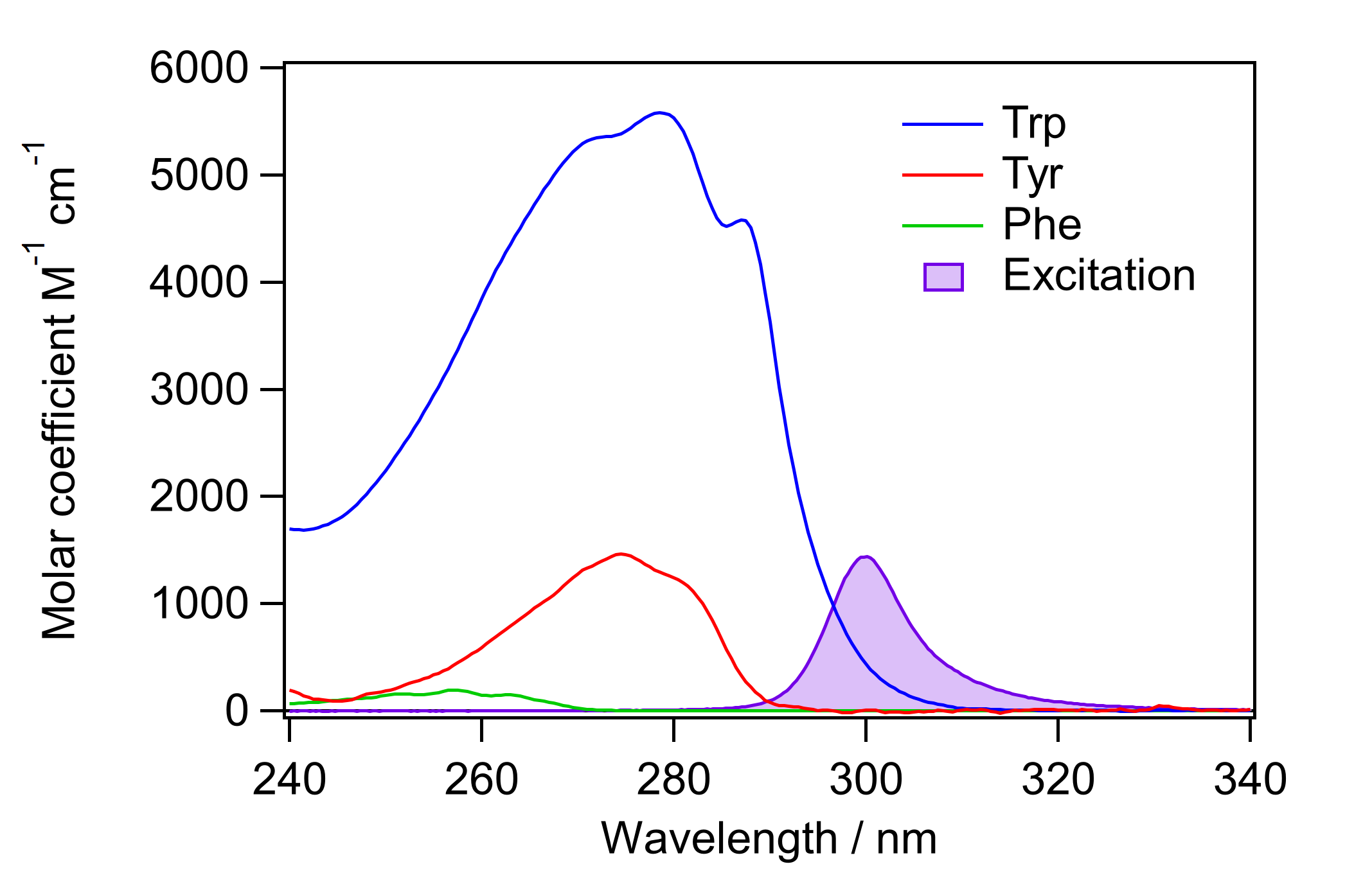


**Figure S3.** Molar coefficient spectra of tryptophan, tyrosine, and phenylalanine. Peak values are ε_278_ = 5580, ε_274_ = 1405, and ε_258_ = 195 M^–1^ cm^–1^, for tryptophan, tyrosine, and phenylalanine, respectively. The spectrum of the UV-pulsed LED light (used as the excitation light) is shown as a purple line.


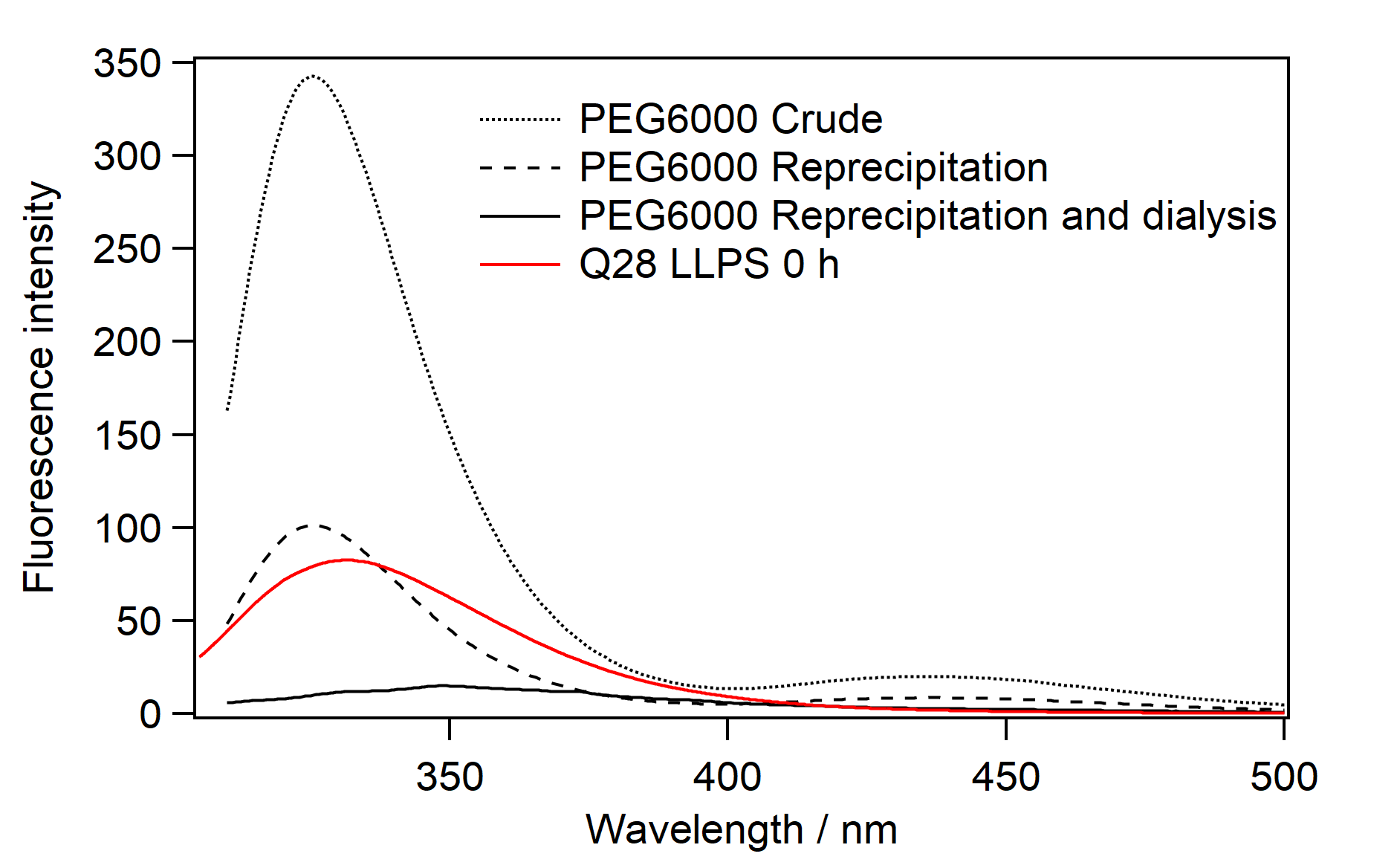


**Figure S4.** Purification of PEG6000. Crude PEG6000 was purchased and purified by reprecipitation and subsequent dialysis. Crude PEG6000 showed fluorescence of impurities at around 325 and 437 nm. These fluorescence signals were largely reduced after reprecipitation and dialysis. Fluorescence spectrum of Q28 immediately after LLPS is shown. The fluorescence signal of the impurities after reprecipitation and dialysis was negligible, compared with that of Q28. The excitation wavelength was 299 nm.


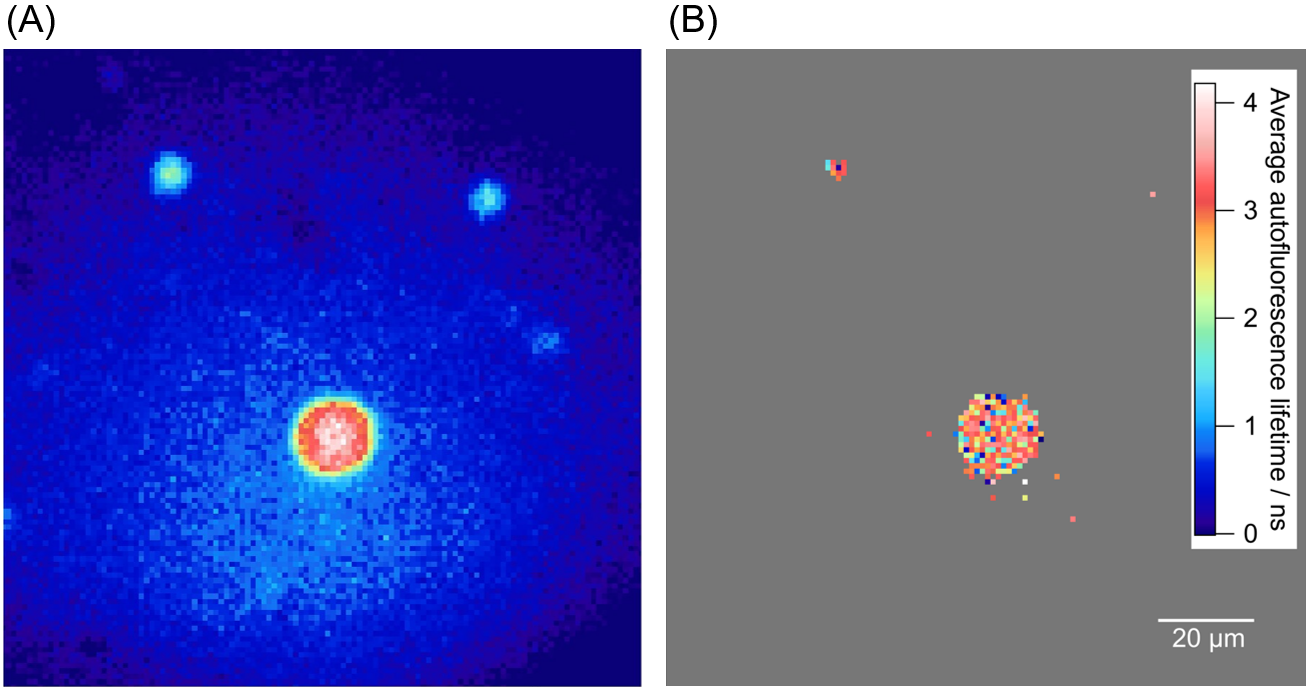


**Figure S5.** Autofluorescence intensity (A) and autofluorescence lifetime (B) images of the Q28 droplets immediately after LLPS. To obtain the lifetime image, we carried out binning of the fluorescence decay curves by 16 pixels, and the obtained traces after 500 ps were fitted with a double exponential function. The average autofluorescence lifetime at each spatial point was then calculated as the sum of the lifetimes of the two components weighted by their pre-exponential factors, and they were plotted in (B). We eliminated the spatial points showing a fluorescence intensity below a threshold value for the lifetime calculation, and they are shown in gray in (B).

**
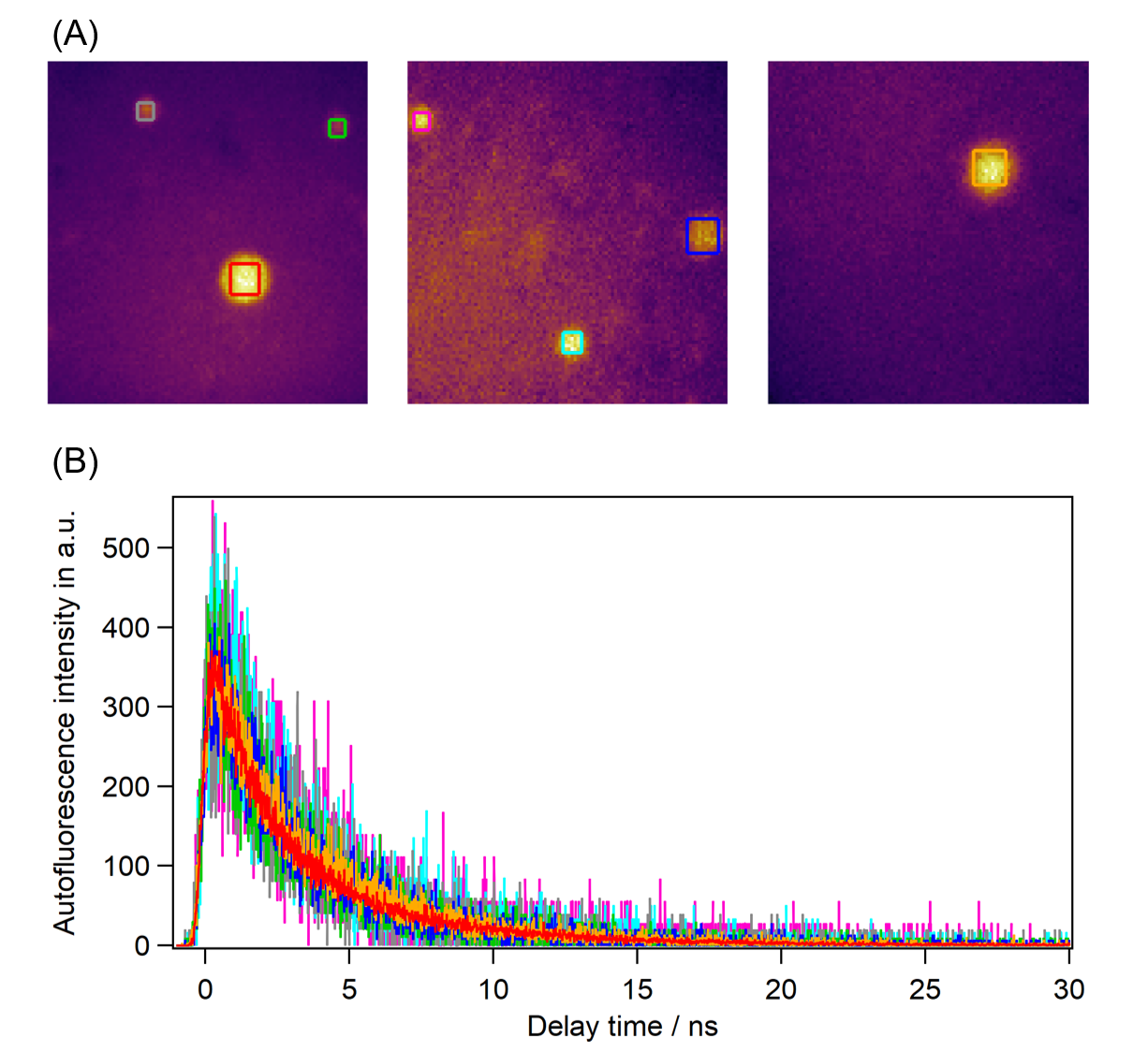
**

**Figure S6.** (A) Autofluorescence images of Q28 droplets. (B) Fluorescence decay curves of individual droplets. Fluorescence decay curves were obtained by summing up the decay curves at spatial points bounded by boxes of the corresponding colour shown in (A).


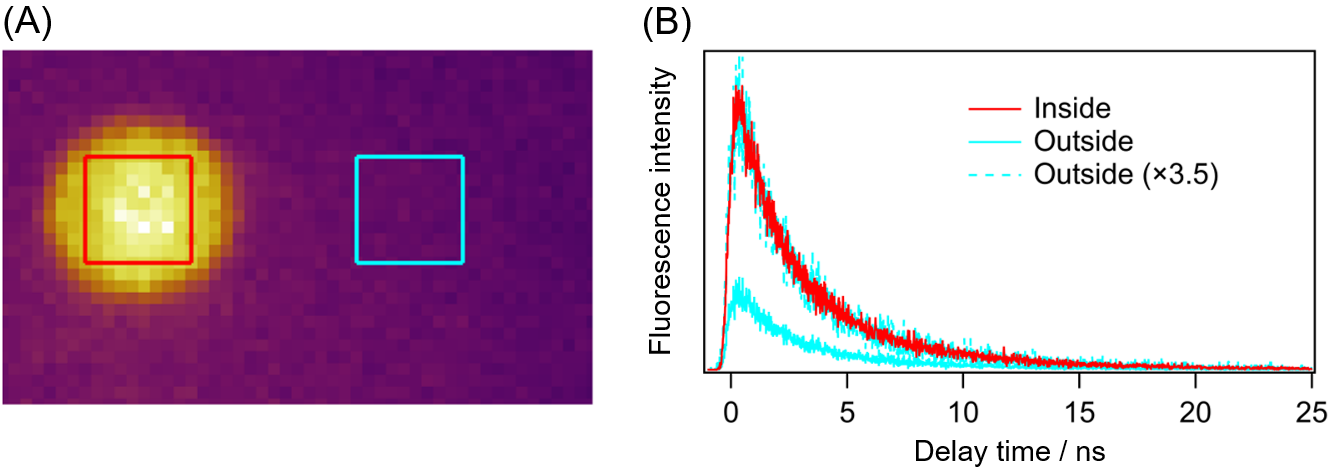


**Figure S7.** Comparison of fluorescence decay curves inside and outside the droplet of Q28. (A) A fluorescence intensity image of the Q28 droplet. Fluorescence decay curves at 100 spatial points surrounded by red and blue boxes in (A) were averaged to construct the fluorescence decay curves inside and outside the droplet, and they are shown in (B). The fluorescence decay curve outside the droplet was multiplied by 3.5 to compare it with that inside the droplet.

**
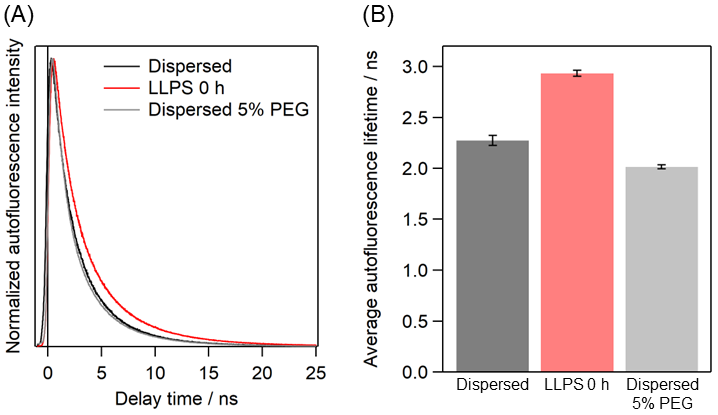
**

**Figure S8.** Comparison of the autofluorescence decay curves (A) and the autofluorescence lifetimes (B) of the droplets and the dispersed solutions with and without 5% PEG. The number of replicates is shown in Table S1.


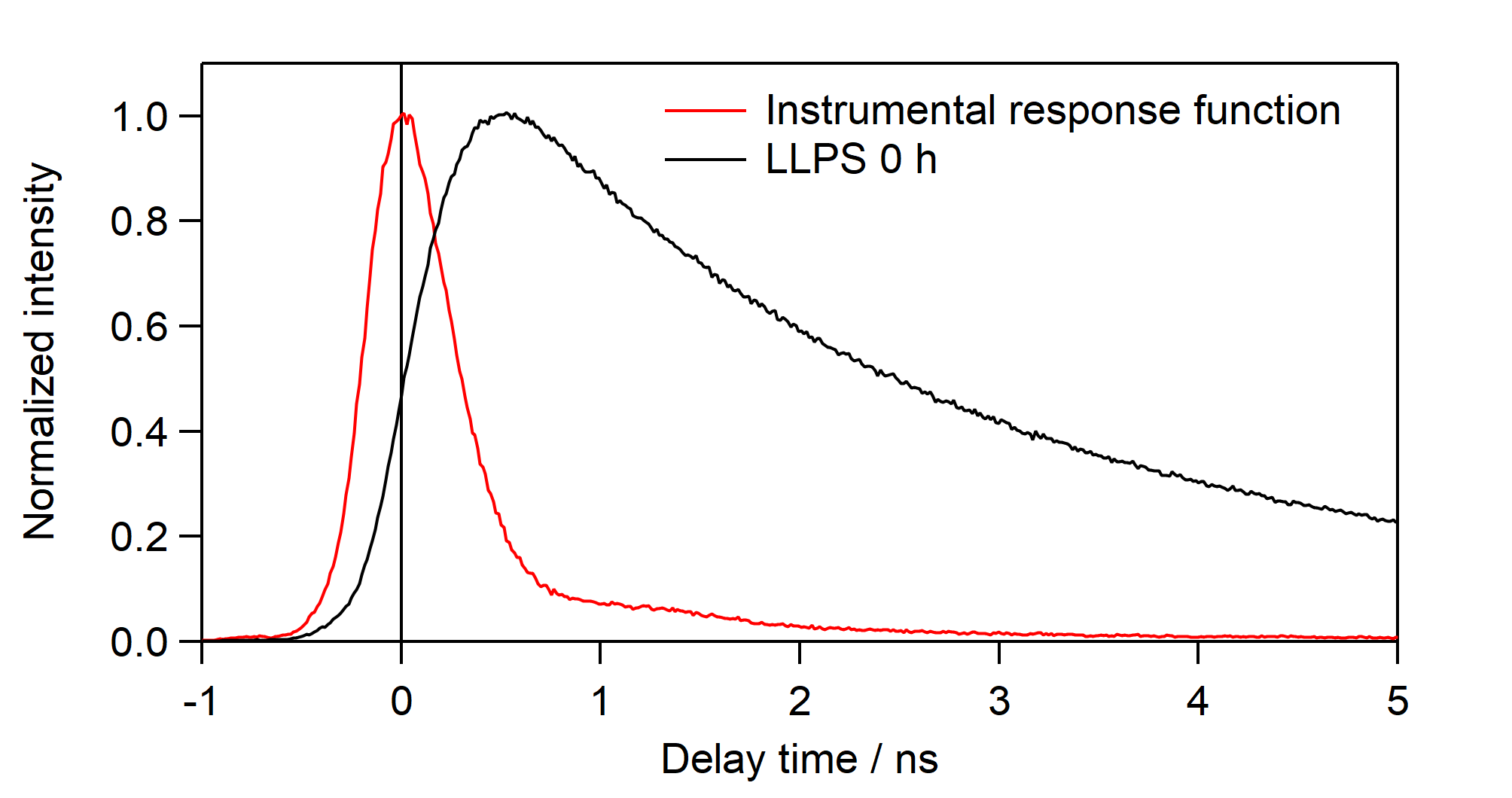


**Figure S9.** A typical instrumental response function in autofluorescence decay measurements (red line), together with the autofluorescence decay curve of Q28 droplets at the incubation time of 0 h (black line).


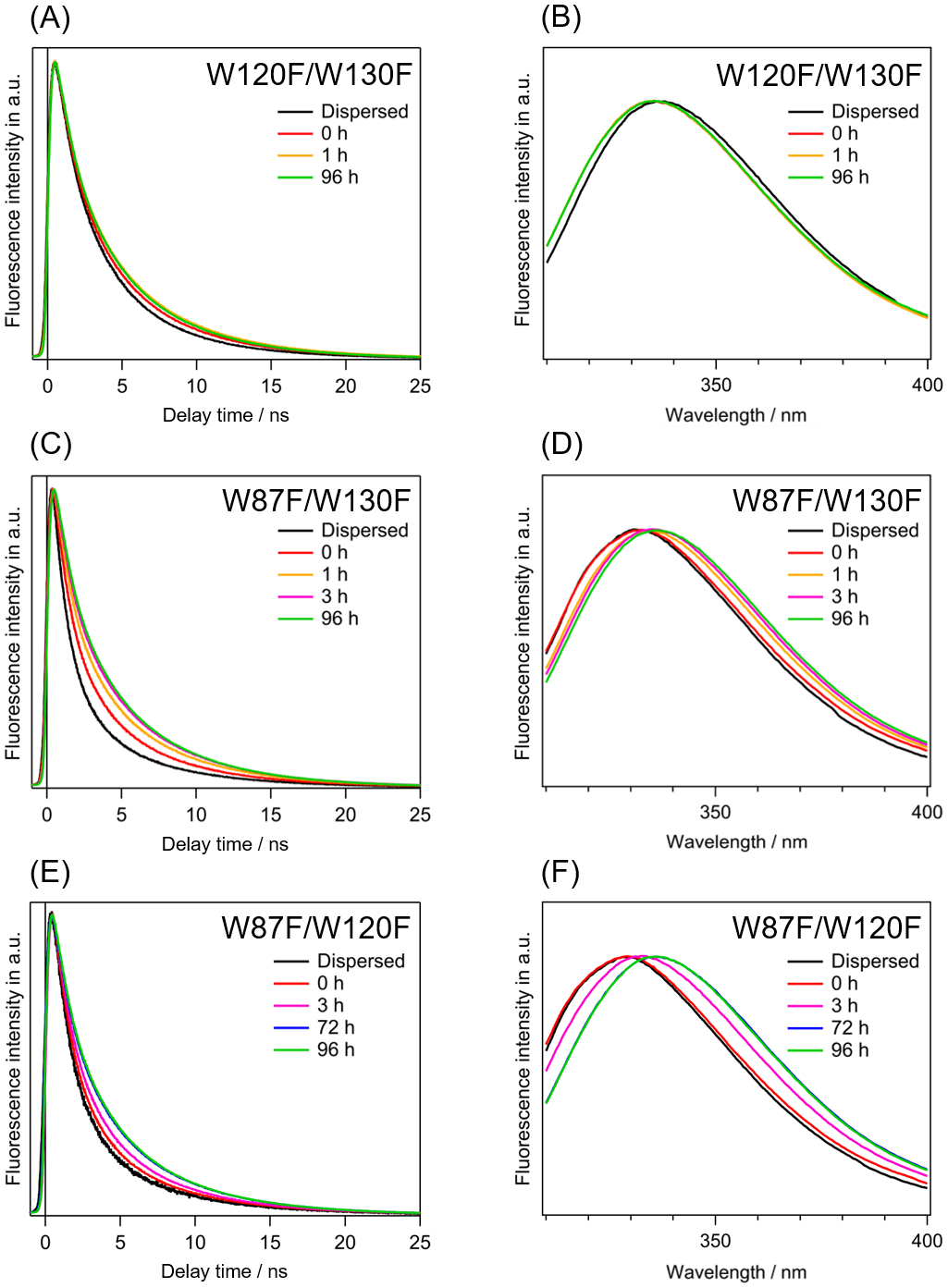


**Figure S10.** Fluorescence decay curves and spectra of dispersed solutions and droplets W120F/W130F, W87F/W130F, and W120F/W130F mutants at various incubation times. The number of replicates is shown in Table S1.


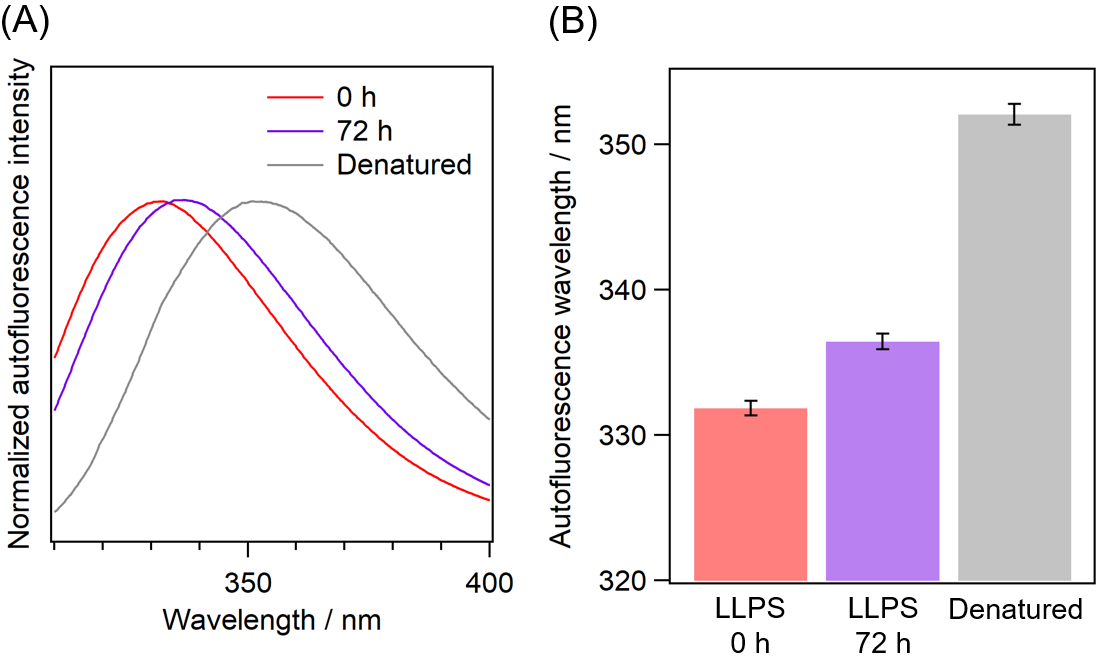


**Figure S11.** Comparison of (A) the autofluorescence spectra and (B) the autofluorescence wavelengths of the Q28 droplets after 0 h and 72 h incubation and Q28 after denaturation with guanidinium chloride. The numbers of replicates are *n* = 7 for LLPS at 0 and 72 h and *n* = 3 for denatured Q28.

**
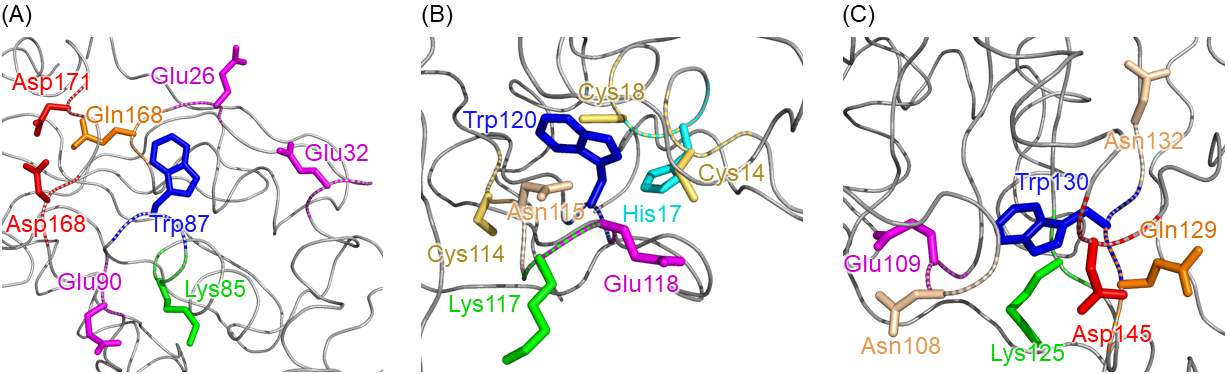
**

**Figure S12.** Amino acid arrangements of ataxin-3 around (A) Trp87, (B) Trp120, and (C) Trp130.

**
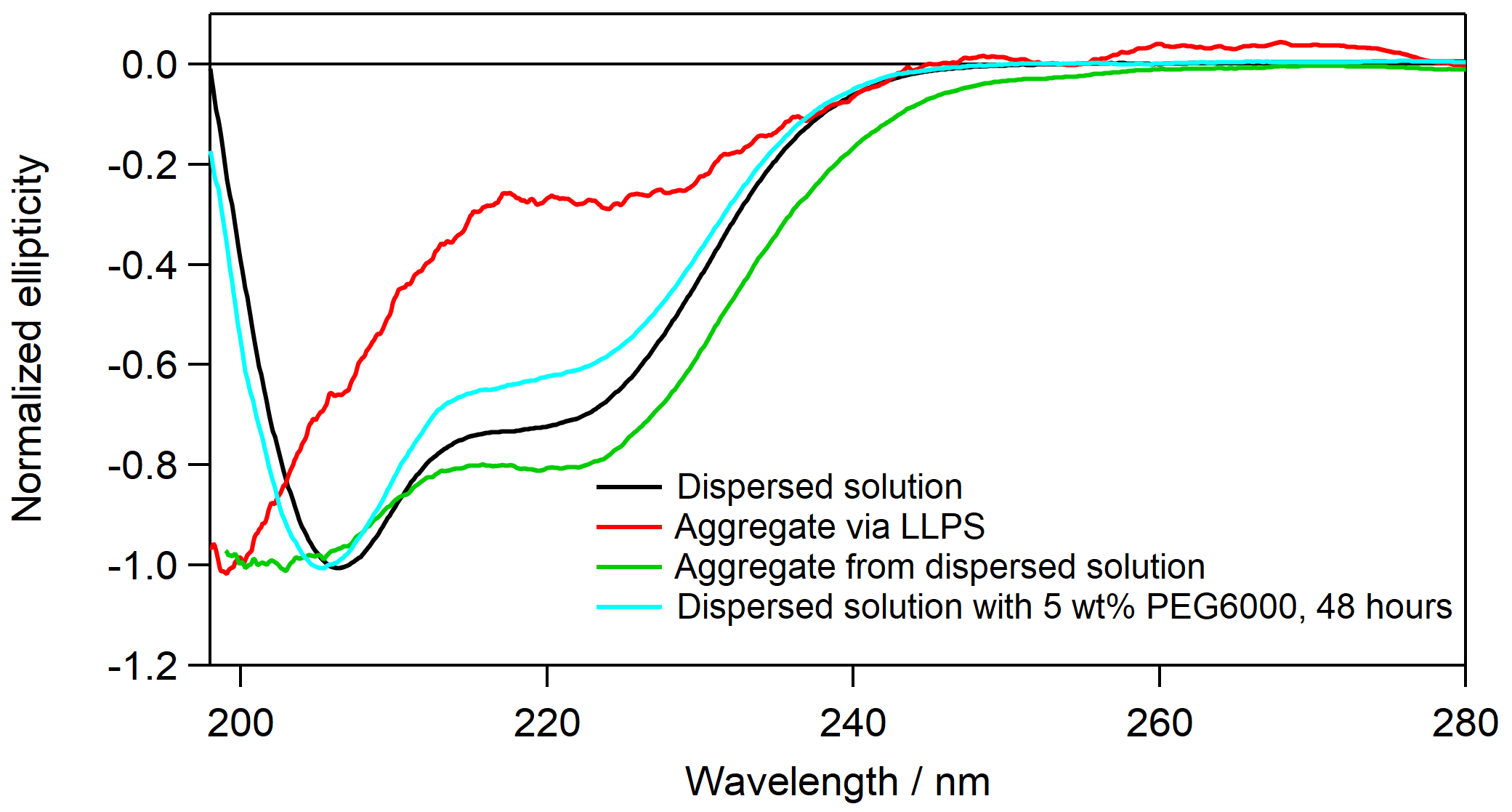
**

**Figure S13.** Circular dichroism (CD) spectra of the dispersed solution (black), the aggregates formed via LLPS (incubated 48 hours at 296 K, red), and those directly formed from the dispersed solution (incubated 10 days at 310 K, green). CD spectrum of the dispersed solution after 48-hour incubation in the presence of 5 wt% PEG6000 is also shown (light blue). The dispersed solution showed negative bands due to α-helix and β-sheet structures at around 220 nm. After the aggregation via LLPS, we observed an increase in the relative amplitude at 200 nm with respect to that at 220 nm, indicating an increase in the random coil content. The spectrum of the aggregate formed from the dispersed solution showed a more pronounced relative amplitude at 220 nm than that formed via LLPS, indicating the presence of secondary structures. The numbers of replicates are *n* = 8, 7, 6, and 3 for the dispersed solution, aggregate via LLPS, aggregate from dispersed solution, and dispersed solution with 5 w/w% PEG6000 after 48 h incubation, respectively.

**
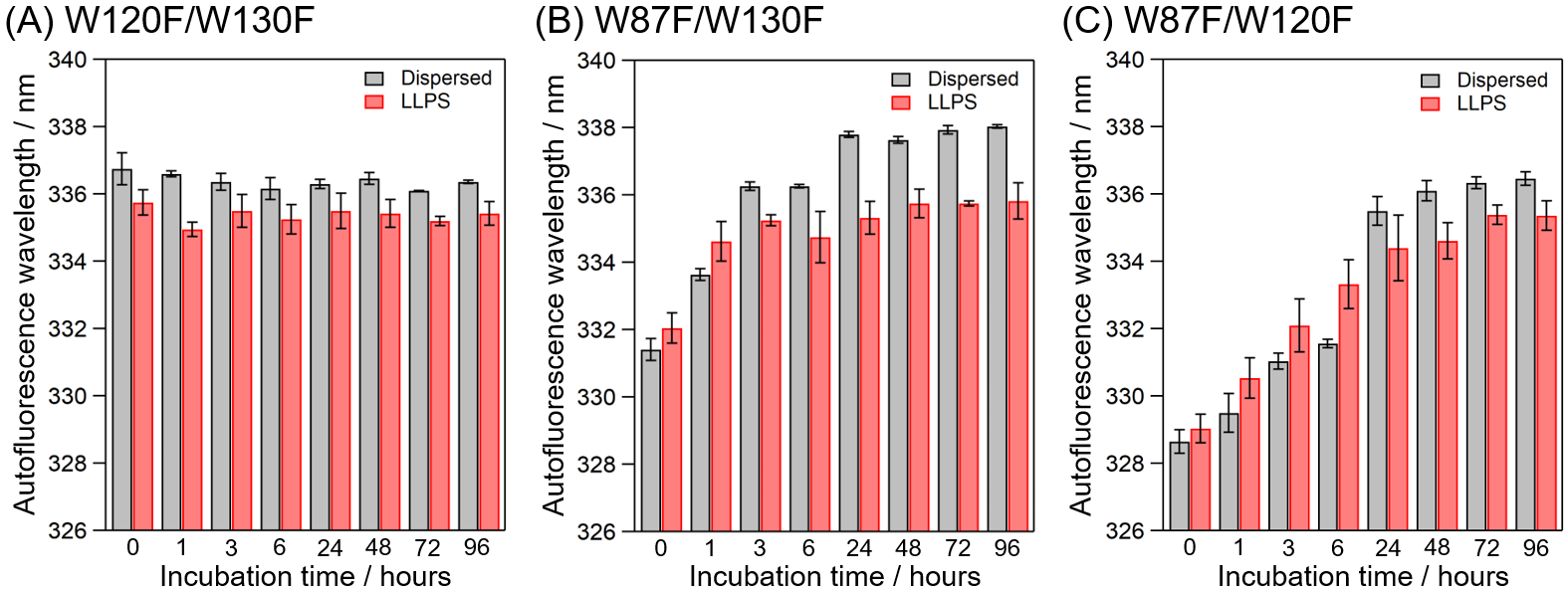
**

**Figure S14.** Fluorescence peak wavelengths of the dispersed solution and the droplets of (A) W120F/W130F, (B) W87F/W130F, and (C) W87F/W120F at various incubation times. The droplets and dispersed solution were incubated at 296 and 310 K, respectively. The wavelengths did not show further changes after 72 h, as shown in (A–C), indicating the completion of the aggregation.

**
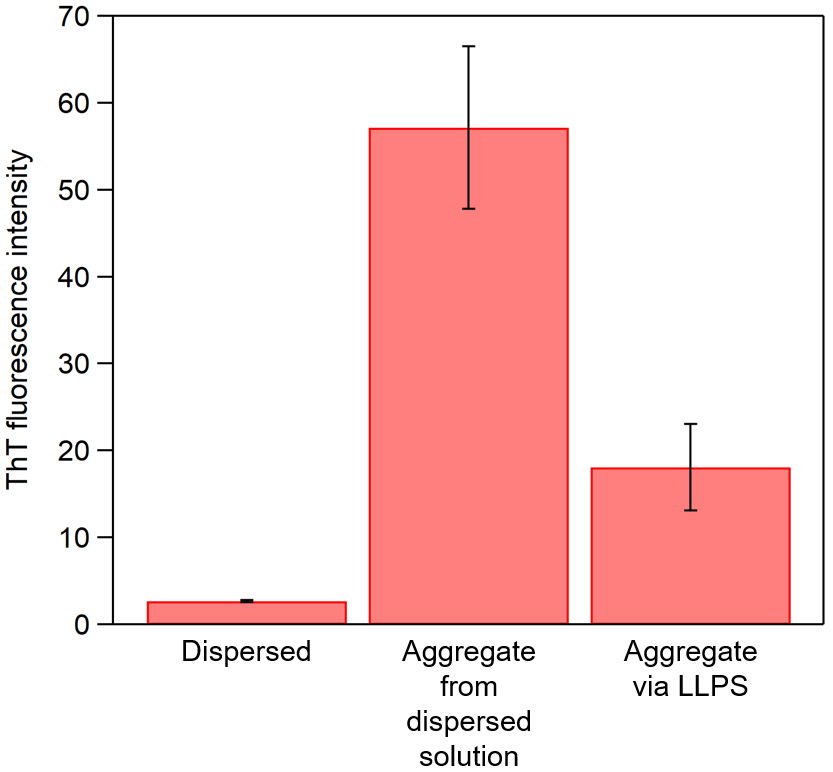
**

**Figure S15.** ThT fluorescence intensities at 480 nm of Q28 in the dispersed solution and aggregates formed directly from the dispersed solution and via LLPS. The ThT fluorescence intensity was largely enhanced by the aggregation from the dispersed solution, indicating the formation of the fibrillar aggregate. The aggregate via LLPS also showed the enhancement of the ThT fluorescence; however, it was markedly weaker than that of the aggregate formed from the dispersed solution. This indicates that the structure of the aggregate formed via LLPS is different from the fibrillar aggregates formed from the dispersed solution. The number of replicates is *n* = 3, 5, and 6 for the dispersed solution, aggregate formed from the dispersed solution, and aggregate formed via LLPS, respectively.


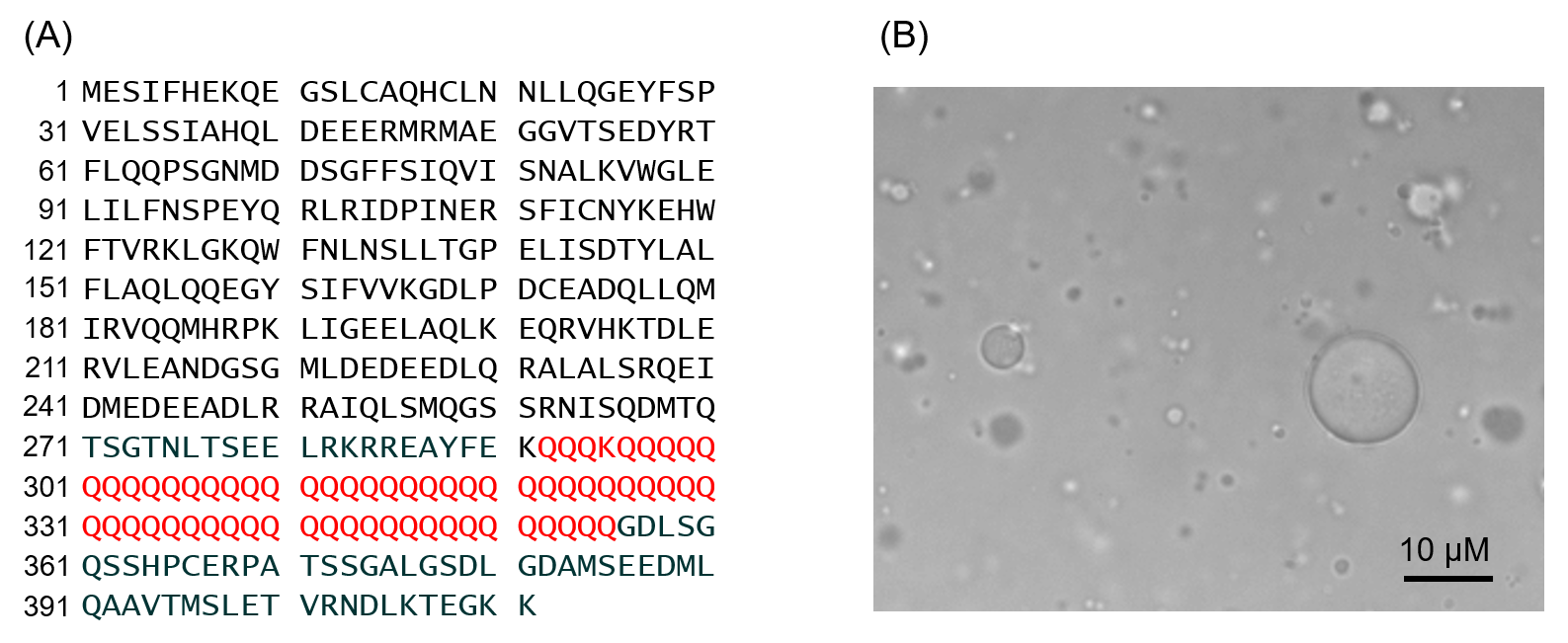


**Figure S16.** (A) Amino acid sequence and (B) LLPS of Q64.

**Table S1.** The number of replicates for evaluating average autofluorescence lifetimes (*τ_ave_*), peak wavelengths (*λ_max_*), and their SDs.

|  |  | Dispersed solution | Droplet | Aggregate |
| --- | --- | --- | --- | --- |
| Figure 2B | *τ_ave_* of Q28 | 3 | 4 | 7 |
| Figure 3B | *λ_max_* of Q28 | 5 | 7 | 7 |
| Figure 5A, S10A, B | *τ_ave_* of Q28 W120F/W130F | 6 | 6 | - |
|  | *λ_max_* of Q28 W120F/W130F | 5 | 4 | - |
| Figure 5B, S10C, D | *τ_ave_* of Q28 W87F/W120F | 6 | 6 | - |
|  | *λ_max_* of Q28 W870F/W120F | 5 | 4 | - |
| Figure 5C, S10E, F | *τ_ave_* of Q28 W87F/W130F | 3 | 6 | - |
|  | *λ_max_* of Q28 W87F/W130F | 5 | 7 | - |
| Figure S8B | *λ_max_* of WT | 7 | 7 | - |

|  | Dispersed solution | 0 h | 1 h | 3 h | 6 h | 24 h | 48 h | 72 h | 96 h |
| --- | --- | --- | --- | --- | --- | --- | --- | --- | --- |
| Figure 7A | 3 | 6 | 6 | 6 | 6 | 6 | 6 | 6 | 6 |
| Figure 7B | 3 | 3 | 3 | 3 | 3 | 3 | 3 | 3 | 3 |

|  | NaCl | KCl | KI | CsCl | NaSCN |
| --- | --- | --- | --- | --- | --- |
| Figure 8 | 5 | 6 | 7 | 3 | 4 |

| Figure S14 | 0 h | 1 h | 3 h | 6 h | 24 h | 48 h | 72 h | 96 h |
| --- | --- | --- | --- | --- | --- | --- | --- | --- |
| W87 dispersed solution | 3 | 3 | 3 | 3 | 3 | 3 | 3 | 3 |
| W87 droplet | 5 | 4 | 4 | 4 | 4 | 4 | 4 | 4 |
| W120 dispersed solution | 3 | 3 | 3 | 3 | 3 | 3 | 3 | 3 |
| W120 droplet | 5 | 4 | 4 | 4 | 4 | 4 | 4 | 4 |
| W130 dispersed solution | 3 | 3 | 3 | 3 | 3 | 3 | 3 | 3 |
| W130 droplet | 5 | 7 | 7 | 7 | 7 | 7 | 7 | 7 |
